# Evidence for peroxisomal redundancy among the glucose-6-phosphate dehydrogenase isoforms of *Arabidopsis thaliana*

**DOI:** 10.1093/pcp/pcaf012

**Published:** 2025-01-18

**Authors:** Loreen Linnenbrügger, Lennart Nico Doering, Louisa-Marlen Tägtmeyer, Kerstin Fischer, Antje von Schaewen

**Affiliations:** University of Münster, Department of Biology, Institute of Plant Biology and Biotechnology, Molecular Physiology of Plants, Schlossplatz 7, Münster 48149, Germany; University of Münster, Department of Biology, Institute of Plant Biology and Biotechnology, Molecular Physiology of Plants, Schlossplatz 7, Münster 48149, Germany; University of Münster, Department of Biology, Institute of Plant Biology and Biotechnology, Molecular Physiology of Plants, Schlossplatz 7, Münster 48149, Germany; University of Münster, Department of Biology, Institute of Plant Biology and Biotechnology, Molecular Physiology of Plants, Schlossplatz 7, Münster 48149, Germany; University of Münster, Department of Biology, Institute of Plant Biology and Biotechnology, Molecular Physiology of Plants, Schlossplatz 7, Münster 48149, Germany

**Keywords:** OPPP, G6PD, dual targeting, peroxisomes, targeting motifs, dimeric protein import

## Abstract

The oxidative pentose phosphate pathway (OPPP) plays an important role in the generation of reducing power in all eukaryotes. In plant cells, the OPPP operates in several cellular compartments, but as full cycle only in the plastid stroma where it is essential. As suggested by our recent results, OPPP reactions are also mandatory inside peroxisomes, at least during fertilization. For the first enzyme of the OPPP, glucose-6-phosphate dehydrogenase (G6PD), we previously showed that one *Arabidopsis* isoform (G6PD1) can be directed to peroxisomes under specific circumstances. Since *g6pd1* knock-out plants are viable, we aimed at elucidating potential redundancy regarding peroxisomal targeting among the other G6PD isoforms. Localization studies of so far cytosolic annotated G6PD5 and G6PD6 (both ending -PTL>) using different reporter fusions of *full-length* versus the last 50 amino acids revealed that GFP-*C-short* versions are efficiently imported into peroxisomes. Modification of the final tripeptide to a canonical peroxisomal targeting signal type 1 (PTS1) also resulted in peroxisomal localization of the *full-length* versions and revealed that G6PD5/6 import may occur as homo- or heterodimer. Interestingly, the new noncanonical PTS1 motif is highly conserved among the cytosolic G6PD isoforms of the Angiosperms, whereas members of the *Poaceae* (rice and maize) possess two variants, one ending with an additional amino acid (-PTLA>) and the other one extended by a stronger PTS1 motif. From both evolutionary and physiological perspectives, we postulate that G6PD import as homo- and heterodimer restricted the acquisition of more efficient peroxisomal targeting motifs to leave some G6PDH activity in the cytosol.

## Introduction

Glucose-6-phosphate dehydrogenase (G6PD) is the initial—and rate-limiting—enzyme of the oxidative pentose phosphate pathway (OPPP). The OPPP comprises three irreversible reactions that catalyse the conversion of glucose 6-phosphate (G6P) to ribulose 5-phosphate (Ru5P), thereby releasing 2 moles of reduced nicotinamide adenine dinucleotide phosphate (NADPH) and 1 mole of carbon dioxide (CO_2_). The irreversible reactions render the OPPP an important metabolic sequence for the generation of reducing power from activated hexoses (reviewed in Kruger and von Schaewen 2003, Esposito 2016). In plant cells, the OPPP mainly operates in plastids at night and in heterotrophic tissues as part of the full pentose phosphate pathway that shares sugar phosphate intermediates with the Calvin-Benson-Bassham cycle and other pathways, like starch biosynthesis (reviewed in Scheibe 1990, Buchanan 1991), as well as the shikimate and linked phenylpropane pathway. The oxidative part (OPPP) on its own occurs either freely in the cytosol (Schnarrenberger et al. 1995), or—as we have recently shown for *Arabidopsis* upon alternative splicing of *G6PD5*—also as membrane-bound metabolon attached to the cytosolic face of the endoplasmic reticulum (ER), where it supports fatty acid elongation and other important membrane-bound pathways that consume NADPH (Linnenbrügger et al. 2022). Ru5P, and upon epimerization xylulose 5-phosphate (Xu5P), are known to enter plastids via members of the triose-phosphate/phosphate translocator family (reviewed in Weber and Linka 2011, Lee et al. 2017, Baune et al. 2020) to support anabolic processes started in or confined to plastids.

After an NADPH recycling system had been found based on G6PDH activity in isolated pea peroxisomes (Corpas et al. 1998), our lab reported that OPPP reactions may be directed to peroxisomes upon cellular redox changes involving thioredoxins that interact with chloroplast-destined precursors in the cytosol, where they function as co-chaperons with either holdase (in reduced state) or foldase (in oxidized state) activity (Meyer et al. 2011, Hölscher et al. 2014). These studies led to an extension of plastidial G6PD classification: aside from P1 enzymes in chloroplasts of photoautotrophic cells/tissues (feedback inhibited by accumulating NADPH levels) and P2 enzymes in plastids of heterotrophic cells/tissues with enhanced NADPH tolerance to enable ferredoxin reduction due to different kinetics (Ki_[NADPH]_ > Km_[NADP]_; Wendt et al. 1999, 2000, Scharte et al. 2009), we elucidated a function for lowly abundant G6PD4 (Wakao and Benning 2005) as founding member of a new catalytically inactive P0 isoform class (Meyer et al. 2011). Importantly, the presence of the two cysteine residues (conserved among all plastidial G6PD isoforms studied so far) rendered G6PD4 still redox-responsive, promoting interaction with G6PD1 in the oxidized state (regulatory disulphide bridge). This was linked to retention of plastidial thioredoxin *m2* (Trx *m2*) in the cytosol during oxidative transients, known to accompany stress and developmental changes (Scharte et al. 2009), leading to interaction of G6PD4 with G6PD1 and peroxisomal import upon exposition of an internal targeting motif within the G6PD1 C-terminus (-SKY-; Meyer et al. 2011).

Although there are other NADPH-regenerating enzymes present in peroxisomes of *Arabidopsis thaliana*, e.g. NADP-isocitrate dehydrogenase (ICDH) that plays a major role in stomata regulation (Leterrier et al. 2016), we could show for the three dually targeted 6-phosphogluconate dehydrogenase (PGD) isoforms (with PGD1 and PGD3 partially residing in the cytosol and plastids) that cytosolic/peroxisomal PGD2 is essential in peroxisomes during fertilization (Hölscher et al. 2016, Doering et al. 2024). The latter suggested a major role for sugar-derived NADPH production by the OPPP inside peroxisomes—at least during this developmental stage.

For the second OPPP step, comprising five 6-phosphogluconolactonase (PGL) isoforms in *Arabidopsis*, we found that dual plastidial/peroxisomal PGL3 (ending -SKL>; Reumann et al. 2007) can also be directed to peroxisomes with the help of Trx *m2* upon retention in the cytosol by an unknown mechanism (Hölscher et al. 2014). Of note, profound effects on genotypic segregation were observed for the *pgl3-1* mutant with altered C-terminal end (lacking a canonical peroxisomal targeting motif, as mentioned later), using reciprocal sibling crosses (Lansing et al. 2020). As described above, among the five catalytically active *Arabidopsis* G6PD isoforms, we could show so far that G6PD1 may enter peroxisomes upon interaction with G6PD4, following a regulatory redox switch involving cytosolic retention of plastidial precursors, including Trx *m2* (Meyer et al. 2011).

Since peroxisomes do not contain their own genome, all proteins with destination peroxisome are nuclear-encoded and have to be imported—also peroxisomal membrane proteins, either via a detour through the ER from where new peroxisomes arise (for review, see van der Zand and Tabak 2013) like peroxisomal ascorbate peroxidase (Bunkelmann and Trelease 1996, Mullen et al. 1999) or directly from the cytosol—like monodehydroascorbate reductase (Leterrier et al. 2005, Lisenbee et al. 2005). For matrix proteins, the two types of peroxisomal targeting signals (PTSs) known for eukaryotes have been studied intensively in yeast versus mammalian cells, and later also in other animal, fungal, or plant species (reviewed in Deb and Nagotu 2017, Pan et al. 2020). PTS type-1 (PTS1) consists of a C-terminal tripeptide, which is recognized by peroxisomal import receptor peroxin 5 (PEX5) in the cytosol, while PTS type-2 (PTS2) consists of a nonameric sequence located within the N-terminus and is bound by peroxin 7 (PEX7). In plant cells, PEX7 binds to PEX5 in the cytosol and then to the pore-forming peroxins (PEX13 and PEX14) for import, followed by redox-regulated cargo release into the matrix (Ma et al. 2013, Apanasets et al. 2014) and recycling of the import receptors back to the cytosol by a conserved protein machinery consisting of several membrane-bound peroxins and an AAA-ATPase complex (reviewed in Pan et al. 2020). The first identified PTS1 tripeptide motif was -SKL> (single-letter code, followed by a stop codon) at the C-terminus of firefly luciferase (Gould et al. 1989). Since then, the tripeptide pattern has expanded and become more diverse, resulting in classification into canonical and noncanonical PTS1 motifs, also for plants (Reumann et al. 2007, Lingner et al. 2011). Moreover, there is cumulating evidence that the upstream region plays an important role as well (Chowdhary et al. 2012). Most recently, approaches using machine learning-based PTS1-prediction algorithms have further increased the number of initially predicted and then experimentally verified PTS1 motifs by about 10-fold (Deng et al. 2024).

Since the report on a *g6pd1* single mutant in the ecotype Landsberg erecta (Siddappaji et al. 2013), we obtained viable *g6pd1 g6pd4* double mutants for the ecotype Columbia (unpublished result of former group member Tanja Meyer), which led us to pursue the question of further redundancy in peroxisomes concerning the remaining catalytically active G6PD isoforms of *A. thaliana*, since NADPH supply in the matrix is needed for several important plant-specific reactions. For instance, maintaining redox balance via the ascorbate-glutathione system and other reactions depending on this reduced cofactor, like l-arginine-dependent nitric oxide synthase activity and certain steps in β-oxidation (for details, see Corpas et al. 2018). More precisely, we aimed at finding hints for cryptic peroxisomal targeting among the cytosolic (G6PD5 and G6PD6) and plastidial P2 isoforms (G6PD2 and G6PD3), which may be able to rescue a presumed fertilization phenotype in the *g6pd1 g6pd4* double-mutant background. By use of fluorescent reporter fusions and confocal laser-scanning microscopy of transfected mesophyll protoplasts, we first showed that the last 50 amino acids (*C-short* versions) of cytosolic G6PD5 and G6PD6 (both ending -PTL>) confer peroxisomal localization. Furthermore, the improvement of peroxisomal import by replacing -PTL> with a canonical PTS1 motif (-SKL> or -SRL>) led to peroxisomal import of the *full-length* versions as well, while deletion of the final leucine from the *C-short* versions resulted in cytosolic patterns, demonstrating that -PTL> is a native, noncanonical PTS1 motif. In addition, we obtained evidence that G6PD5 and G6PD6 may enter peroxisomes as dimers (1st OPPP step), by contrast to our most recent report on PGD2 (3rd OPPP step), for which monomeric import was strongly favoured (Doering et al. 2024). Surprisingly, phylogenetic analyses of cytosolic G6PD homologs with a focus on the green lineage revealed high conservation of the -PTL> motif among the Angiosperms, and beyond that occurrence of two different cytosolic isoforms in members of the *Poaceae*, either extended by one amino acid (resulting in -PTLA>) or by a functional second PTS1 motif (as defined in Chowdhary et al. 2012). Hence, together our results hint at the redundancy of different G6PD isoforms in peroxisomes and important reasons for retaining a relatively weak PTS1 motif in the cytosolic isoforms of most higher plant species.

## Results

### The last 50 amino acids of the cytosolic G6PD isoforms confer peroxisomal import

This study was undertaken due to the occurrence of viable *Arabidopsis g6pd1* (Siddappaji et al. 2013) and *g6pd1 g6pd4* double-mutant plants (Meyer T, unpublished work) plus our findings regarding the third OPPP step that pointed to a need for PGD2 in peroxisomes during fertilization (Hölscher et al. 2016, Doering et al. 2024). Thus, the main question was whether one of the other catalytically active G6PD isoforms could possibly enter peroxisomes. We first analysed the two cytosolic isoforms, i.e. splice variants G6PD5.1 and G6PD6 (Linnenbrügger et al. 2022), since both proteins end -PTL>, a tripeptide that does not match the known consensus sequences (x[KR][LMI]>, [SA]y[LMI]>, or [SA] [KR]z>) of canonical PTS1 motifs (Chowdhary et al. 2012), but was recently found to target peroxisomes when C-terminally fused to an auxiliary peptide sequence that is N-terminally attached to a fluorescent reporter (Deng et al. 2024). Localization studies in *Arabidopsis* mesophyll protoplasts using *full-length* G6PD5.1 and G6PD6 (N-terminally fused to GFP) showed only a cytosolic localization pattern and no colocalization with peroxisomal markers, i.e. OFP-PGL3*_C-short* (last 50 amino acids of PGL3; Meyer et al. 2011) or membrane marker PEX16-OFP (Baune et al. 2020).

**Figure 1.**
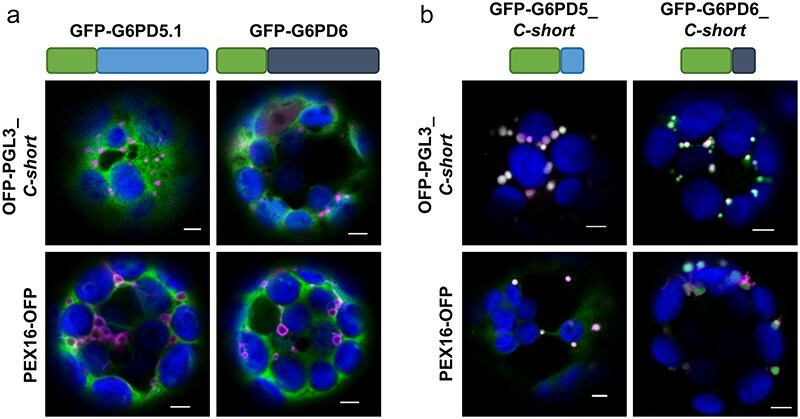
The last 50 amino acids of the cytosolic *Arabidopsis* G6PD isoforms confer peroxisomal targeting. Coexpression in *Arabidopsis* wild-type (Col-0) mesophyll protoplasts. (a) G6PD5.1 and G6PD6 *full-length* or (b) only the part coding for the last 50 amino acids (ending -PTL>) N-terminally fused to GFP (schematically depicted above) with two different peroxisomal markers: top panels, soluble marker OFP-PGL3_*C-short* (the last 50 amino acids of PGL3 ending -SKL>); bottom panels, membrane marker PEX16-OFP. Note that the signals of the GFP-G6PD *full-length* fusions are evenly distributed in the cytosol, showing no overlap with the peroxisomal markers. Only the corresponding G6PD_*C-short* versions accumulate in dot-like structures that colocalize with both markers (bright dot-like signals). The images show single optical sections as the merger of all fluorescent channels (for single-channel images, see Supplementary Fig. S1). GFP is depicted in green, OFP in magenta, and chlorophyll autofluorescence in blue; white signals indicate colocalization or very close signals (<200 nm) of GFP and OFP. Scale bars, 3 μm.

Thus, since short C-terminal fusions (∼50 amino acids) usually suffice and are favourable over shorter versions concerning peroxisomal localization studies (Hölscher et al. 2014), the last 50 amino acids of the cytosolic G6PD isoforms were cloned behind monomeric GFP and coexpressed with the peroxisomal markers. Coexpression with the matrix marker OFP-PGL3_C-*short* (ending -SKL>) resulted in a complete overlap of the GFP and OFP (orange-shifted monomeric red fluorescent protein mRFP) signals in round structure sizes of 1–2 µm, and similar GFP-labelled structures were surrounded by OFP signals of the peroxisomal membrane marker (PEX16-OFP; Fig. 1b). This demonstrated that peroxisomal import is efficiently mediated by the last 50 amino acids of G6PD5.1 and G6PD6, both ending -PTL>.

### Medial reporter fusions of the cytosolic G6PD isoforms are catalytically active

**Figure 2.**
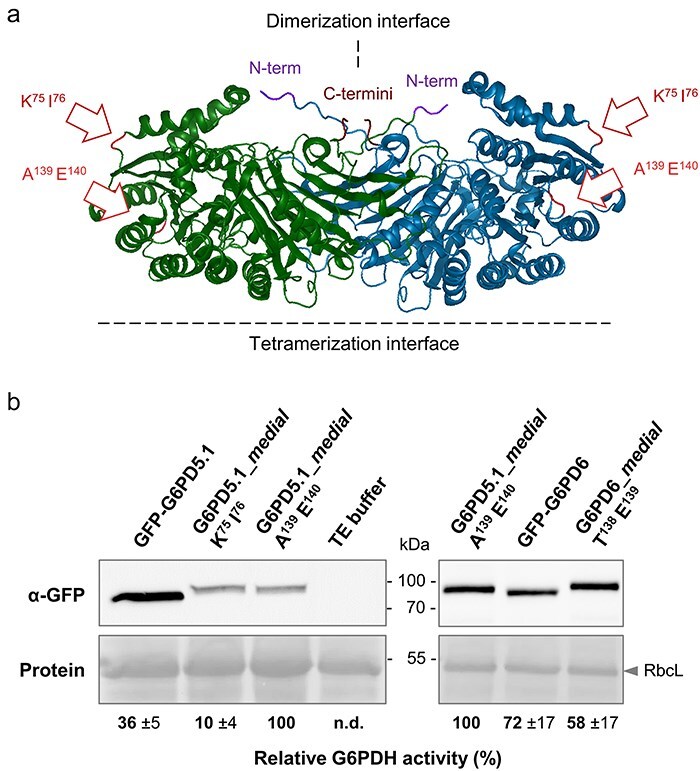
One of two G6PD5.1_*medial* GFP fusions proved to be highly active. (a) G6PD5 dimer (PDB file obtained from Alphafold, modified with Protean 3D), with dimerization and tetramerization interfaces (dashed lines) based on human G6PD enzymes (Supplementary Fig. S2). The positions selected for insertion of the GFP reporter into G6PD5 lie in outer loop regions (after 75 and 139 amino acids, respectively), as indicated by white arrows with red outlines. Note that 3D structure prediction for the N-terminus (N-term) is weak. (b) Immunoblot analysis of protein extracts prepared from *g6pd5-1 g6pd6* double-mutant protoplasts upon transfection with the indicated GFP constructs or TE buffer (control), using anti-GFP antibodies (α-GFP). Protein refers to signals on the Ponceau S-stained blot with RubisCO large subunit (RbcL) as loading reference. G6PDH activity was determined ∼24 h post-transfection with at least three measurements per extract from three independent experiments. Relative activities were calculated (±SD) based on corresponding band intensities on the α-GFP immunoblots, with G6PD5.1_*medial* (A^139^ E^140^) set to 100%. Abbreviation: n.d., not detectable.

To overcome a possible interference of the *full-length* fusions with dimerization issues or interaction partners at the N-terminus, *medial* GFP-reporter fusions were cloned. Based on the 3D structure of human G6PD with known dimerization and tetramerization interfaces, two possible integration sites were selected for GFP insertion into the cytosolic plant enzymes (Fig. 2a). To test whether the *medial* GFP fusions are active, protoplasts were prepared from leaves of *g6pd5 g6pd6* double-mutant plants that do not exhibit detectable G6PDH activity (Linnenbrügger et al. 2022). Aliquots were transfected with different reporter fusions (or TE buffer as negative control) and harvested 24 h after transfection. All constructs are driven by the strong constitutive 35S promoter of cauliflower mosaic virus, which resulted in measurable G6PDH activity levels. Band intensities of the same extracts upon immunoblot development with anti-GFP antibodies served for quantification of the corresponding expression levels of the fusion proteins. G6PD5.1_*medial* (A^139^ E^140^) turned out to be the one with highest catalytic activity, wherefore it was set to 100% (Fig. 2b). In comparison, G6PD5.1_*medial* (K^75^ E^76^) showed only 10% and the *m*GFP-G6PD5.1 fusion 36% relative activity, stressing the benefit of testing several *medial* reporter fusions (Fig. 2b, left).

The better insertion site was also used for G6PD6 (between amino acid T^138^ E^139^). leading to 58% relative activity compared to G6PD5.1_*medial* (A^139^ E^140^) and 72% relative activity compared to the C-terminal *m*GFP-G6PD6 fusion (Fig. 2b, right). Considering the relatively high standard deviations (±17%), we assume comparable activity for both G6PD6 fusions. In any case, we obtained *medial* reporter fusions that are still able to form active dimers—in the following referred to as G6PD5.1_*medial* and G6PD6_*medial*.

### Peroxisomal import of G6PD5.1 and G6PD6 depends on the PTS1 motif

**Figure 3.**
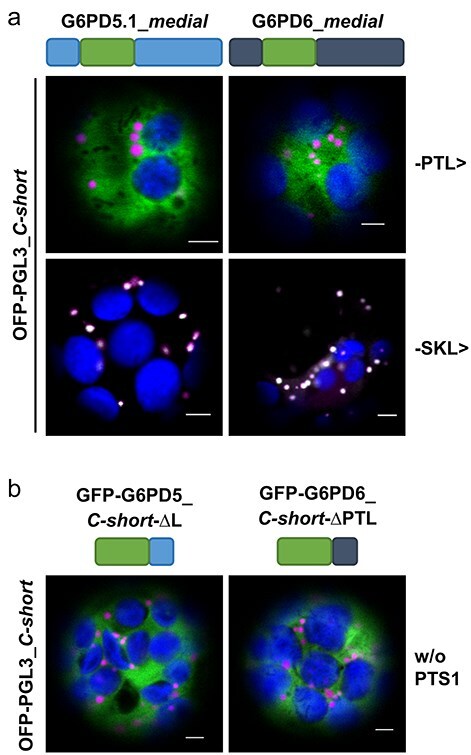
Peroxisomal import of G6PD5 and G6PD6 depends on a C-terminal PTS1 motif. Coexpression in *Arabidopsis* wild-type (Col-0) mesophyll protoplasts. (a) The new *medial* GFP fusions of G6PD5.1 and G6PD6 (schematically depicted above) ending either -PTL> (top) or with canonical PTS1 motif -SKL> (bottom) like the soluble peroxisomal marker (OFP-PGL3_*C-short* ending -SKL>). While the original versions show no overlap with the OFP signals, the engineered -SKL> versions show colocalization (bright signals) in dot-like structures. (b) Coexpression with the corresponding *C-short* constructs (schematically depicted above) lacking a PTS1 motif, either upon deletion of only the last amino acid (ΔL) or the entire PTS1 motif (ΔPTL). Note that both result in a cytosolic localization pattern. The images show single optical sections as the merger of all fluorescent channels (for single-channel images, see Supplementary Fig. S3). GFP is depicted in green, OFP in magenta, and chlorophyll autofluorescence in blue; white signals indicate colocalization or very close signals (<200 nm) of GFP and OFP. Scale bars, 3 μm.

Next, we transfected *Arabidopsis* wild-type protoplasts to investigate the localization of the *medial* GFP fusions. Coexpression with the peroxisomal matrix marker (OFP-PGL3_*C-short*) resulted in a cytosolic localization pattern (Fig. 3a, top), similar to the N-terminal fusions (Fig. 1a). To check if peroxisomal import may be achieved by a stronger canonical PTS1 motif, the C-terminal -PTL> was exchanged for -SKL> in both *medial* reporter constructs, which resulted in clear colocalization with the peroxisomal matrix marker (Fig. 3a, bottom). We also coexpressed both variants with the peroxisomal membrane marker (PEX16-OFP) to double check localization of the -PTL> constructs and found GFP signal inside peroxisomes only for the -SKL> variants (Supplementary Fig. S4A). Furthermore, we confirmed that the exchange of the C-terminal tripeptide (from -PTL> to -SKL>) did not abolish catalytic activity (Supplementary Fig. S5). Additionally, we tested -SRL> instead of -SKL> to exclude the possibility of PTS1 weakening by post-translational modification (i.e. charge-neutralizing lysine acetylation; Finkemeier et al. 2011), which resulted in similarly efficient import of the *medial* constructs (Supplementary Fig. S4B). Together, these findings demonstrate the potential of the G6PD5.1 and G6PD6 proteins to enter peroxisomes.

To further investigate the role of the C-terminal tripeptide -PTL>, we mutagenized the motif in the G6PD5.1 and G6PD6_*C-short* constructs. Deletion of the last leucine (GFP-G6PD5_*C-short*-ΔL) already changed the pattern to sole cytosolic GFP signal, showing no more colocalization with the peroxisomal marker (Fig. 3b, left). Correspondingly, deletion of the entire motif from G6PD6 (GFP-G6PD6_*C-short*-ΔPTL) resulted in a similar cytosolic localization pattern (Fig. 3b, right), underlining that the last three amino acids exhibit PTS1 function.

### Evidence for peroxisomal import of G6PD homo- and heterodimers

**Figure 4.**
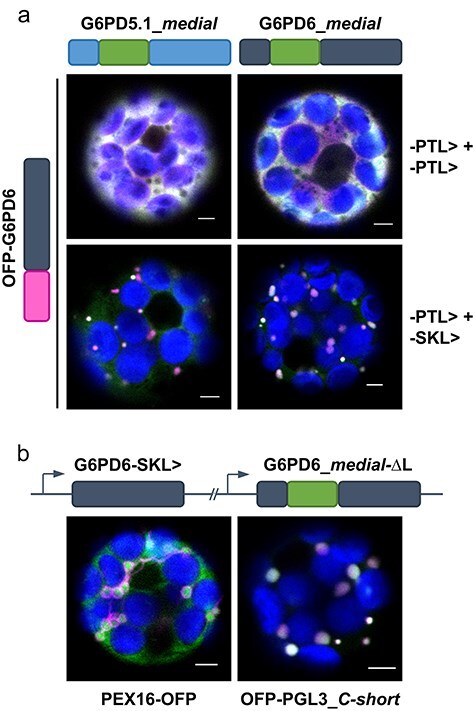
Peroxisomal import of G6PD5 and G6PD6 may occur as hetero- or homo-dimer. Coexpression in *Arabidopsis* wild-type (Col-0) mesophyll protoplasts: (a) The *medial* GFP fusions of G6PD5.1 (left) and G6PD6 (right, both ending -PTL>) were coexpressed with OFP-G6PD6 ending either -PTL> (top) or with engineered canonical PTS1 motif -SKL> (bottom). In both cases of the latter, a clear change in colocalization (white signals) occurred. (b) Localization study using a double cassette construct, containing G6PD6 ending -SKL> (without fluorescent reporter) and the G6PD6_*medial* GFP fusion without the last amino acid (ΔL, to destroy the PTS1 motif), upon coexpression with the peroxisomal membrane marker PEX16-OFP (left) or soluble peroxisomal marker OFP-PGL3_*C-short* (right). In both cases, dot-like accumulations of the GFP signal were observed, either surrounded by OFP (left) or colocalizing with OFP (white signals, right). The images show single optical sections as the merger of all fluorescent channels (for single-channel images, see Supplementary Fig. S6). GFP is depicted in green, OFP in magenta, and chlorophyll autofluorescence in blue; white signals indicate colocalization or very close signals (<200 nm) of GFP and OFP. Scale bars, 3 μm.

Next, we investigated the possibility of dimeric protein import and coexpressed either G6PD5.1 or G6PD6 as *medial* GFP fusions with N-terminally OFP-labelled G6PD6 ending either -PTL> or -SKL>. Coexpression of both -PTL> versions resulted in colocalization in the cytosol (white signals; Fig. 4a, top). Yet, when one protein carried the -PTL> and the other the stronger PTS1 motif -SKL>, colocalization was confined to small round accumulations (Fig. 4a, bottom). Since the -SKL> version of G6PD6 was able to change the localization pattern of wild-typical G6PD5.1 with -PTL> motif, we concluded that the cytosolic G6PD isoforms may form homo- or heterodimers that are imported into peroxisomes.

To check, whether the accumulations occur inside peroxisomes and if this is really due to the stronger PTS1-carrying dimer part, a double cassette construct was created with unlabelled G6PD6 ending -SKL> and the *medial* GFP fusion of G6PD6 lacking the last Leucine, thus destroying the native PTS1 motif (shown in Fig. 3b for G6PD5_*C-short*-ΔL). Coexpression of this construct with either of the two peroxisomal markers confirmed peroxisomal localization of the PTS1-lacking *medial* GFP fusion (Fig. 4b). These results indicate that G6PD6, and most likely G6PD5.1 as well, can be imported into peroxisomes as dimers, even if one part is missing a proper PTS1 motif.

These results are further supported by coexpression of the G6PD5 and G6PD6 *C-short* constructs with or without a PTS1 motif, which did not show colocalization in peroxisomes (Supplementary Fig. S7). Hence, G6PD versions of only 50 amino acids are unable to dimerize and thus imported as monomers.

### Not every *C-short* version or full-length protein ending -SRL> is imported by peroxisomes

**Figure 5.**
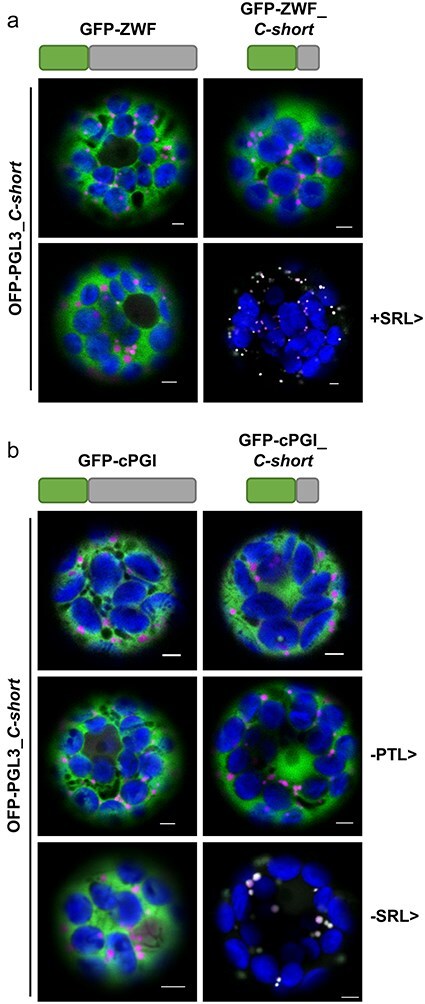
Peroxisomal import of G6PD variants is governed by more than the last three amino acids. Coexpression of the soluble peroxisomal marker (OFP-PGL3_*C-short*, ending -SKL>) in *Arabidopsis* wild-type (Col-0) mesophyll protoplasts, (a) with the G6PD homolog of *E. coli* (ZWF, ‘*Zwischenferment*’, ending -EFE>) fused to GFP (left), or only the last 50 amino acids (right). Wild-type versions (top panels), plus canonical PTS1 motif -SRL> (bottom panels). (b) Coexpression of OFP-PGL3_*C-short* with cPGI (ending -PQM>) fused to GFP (left), or only the last 54 amino acids (right). Wild-type versions (top panels), or similar to G6PD5 and G6PD6 with engineered -PTL> (centre panels), and with canonical PTS1 motif -SRL> (lower panels). Note that only the short versions with additional or engineered canonical PTS1 motif colocalize with the peroxisomal marker. The images show single optical sections as the merger of all fluorescent channels (for single-channel images, see Supplementary Fig. S8). GFP is depicted in green, OFP in magenta, and chlorophyll autofluorescence in blue; white signals indicate colocalization or very close signal (<200 nm) of GFP and OFP. Scale bars, 3 μm.

During the course of our study, we found several examples demonstrating that not every *short* and not every *full-length* version with a PTS1 motif may localize in peroxisomes. For example, the G6PD of *Escherichia coli*—termed ZWF, like in yeast (for ‘*Zwischenferment*’ coined by Otto H. Warburg; Horecker 1976)—was N-terminally fused to GFP and equipped with a canonical PTS1 motif (-SRL>). Coexpression with the peroxisomal matrix marker showed no colocalization (neither for the native variant) (Fig. 5a, left), even though the last 50 amino acids of ZWF with added -SRL> did (Fig. 5a, right). This demonstrated that not every G6PD dimer can be imported by peroxisomes.

In another approach, we used cytosolic glucose-6-phosphate isomerase (cPGI) regarding the possibility of direction to peroxisomes, since its native C-terminal end (-PQM>) was listed by the new machine-learning algorithm and had been experimentally confirmed, similar to -PTL> (Deng et al. 2024). Yet, neither the GFP-cPGI *full-length* nor the *C-short* version showed colocalization with the peroxisomal matrix marker (Fig. 5b, top). The same localization pattern was observed when the terminal tripeptide of cPGI was changed to -PTL> (Fig. 5b, centre). When changed to -SRL>, only the *C-short*, but not the *full-length* GFP-cPGI version colocalized with the peroxisomal marker (Fig. 5b, bottom). These findings demonstrate that a strong canonical motif alone does not guarantee that a GFP *full-length* fusion enters peroxisomes, likely since the PTS1 upstream region and other regulating mechanisms or factors (e.g. interaction partners) may play an additional role.

### The -PTL> motif is highly conserved among Angiosperm homologs

**Figure 6.**
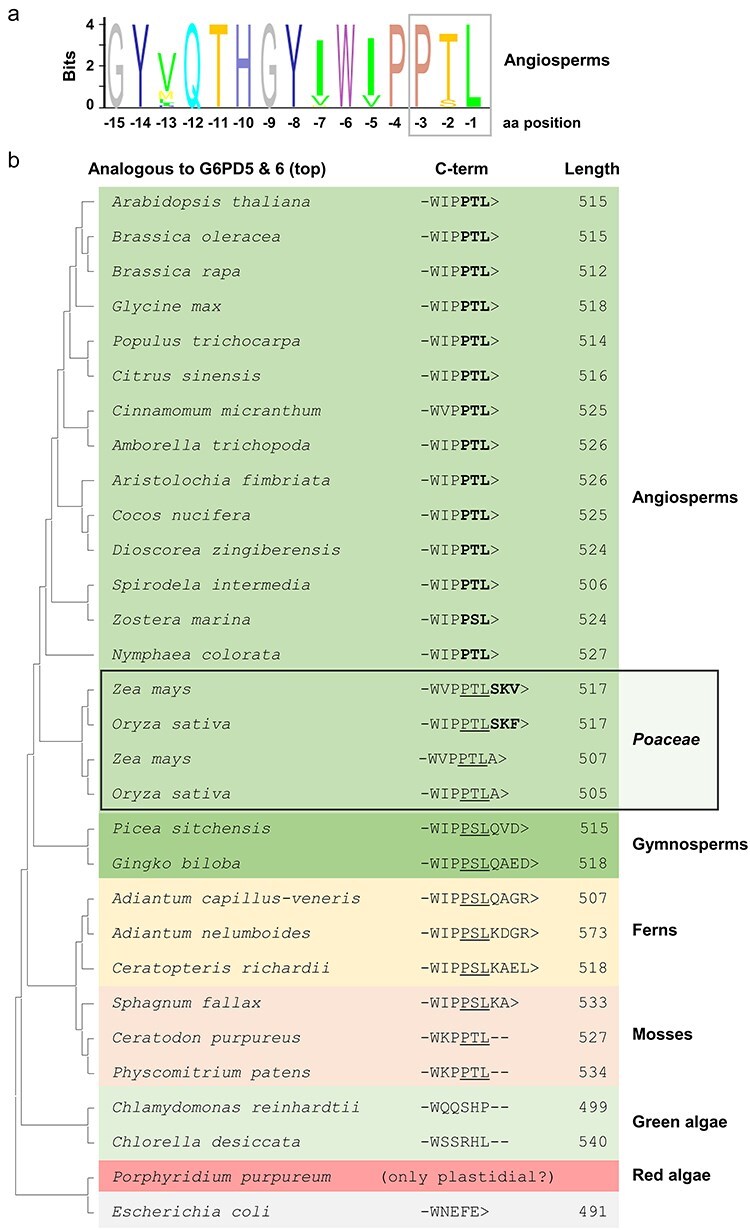
Phylogenetic analyses hint at a conserved function of weak PTS1 signal -PTL> in the Angiosperms. (a) ‘Sequence logo’ analysis (created with MATLAB) of the last 15 amino acids of AtG6PD6 homologs derived from 41 Angiosperm species, showing high conservation of the novel PTS1 motif -PTL>. (b) The maximum likelihood tree of AtG6PD6 homologs selected from different species was created with Mega (see also Supplementary Fig. S9). Subtrees were flipped to present *A. thaliana* on top. The C-terminal 6–9 amino acids highlight conservation of the PTL motif in the Angiosperm sequences with exceptions in the grasses (*Poaceae*) and PSL or PTL occurring internally in the sequences of ferns and mosses (motifs underlined).

With regard to a potential influence of the PTS1 upstream region, we investigated G6PD6 homologs in different species of the green linage. Among the angiosperms, the last 15 amino acids turned out to be highly conserved (Fig. 6a) including the C-terminal -PTL> tripeptide, with only the seagrass *Zostera marina* ending -PSL> (Fig. 6b).

**Figure 7.**
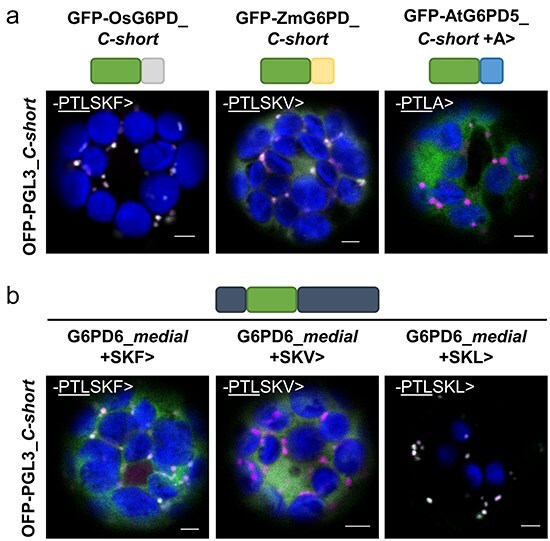
Isoforms of the *Poaceae* rice and maize could also be located inside peroxisomes. (a) Coexpression of *C-short* constructs of G6PD isoforms from rice (Os, *O. sativa*, left panels), maize (Zm, *Z. mays*, centre panels), and AtG6PD5 with additional alanine (+A, right panels) in *Arabidopsis* Col-0 mesophyll protoplasts with the soluble peroxisomal marker. Note that the short rice and maize fusions show peroxisomal localization, while the AtG6PD5 version ending -PTLA> labels the cytosol. (b) Coexpression of G6PD6_*medial* ending either like rice (-PTLSKF>, left panels), maize (-PTLSKV>, centre panels), or with attached canonical PTS1 motif (-PTLSKL>, right panels). Marker, soluble OFP-PGL3_*C-short* (ending -SKL>). The images show single optical sections as the merger of all channels (for single-channel images, see Supplementary Fig. S10). GFP is depicted in green, OFP in magenta, and chlorophyll autofluorescence in blue; white signals indicate colocalization or very close signals (<200 nm) of GFP and OFP. Scale bars, 3 μm.

Interestingly, among the grasses, *Oryza sativa* and *Zea mays* possess two cytosolic G6PD isoforms, ending either -PTLA> or with a double PTS1 motif (-PTLSKF> and -PTLSKV>, respectively; Fig. 6b, black box). We cloned the corresponding GFP *C-short* versions and tested them in *Arabidopsis* mesophyll protoplasts. Of note, both colocalized with the peroxisomal markers, whereas a *C-short* construct of AtG6PD5 ending -PTLA> did not (Fig. 7a). In addition, we cloned G6PD6_*medial* fusions ending like *O. sativa* (PTLSKF>) or *Z. mays* (-PTLSKV>) to check for localization of these *full-length* versions in comparison to a variant ending with a canonical PTS1 (-PTLSKL>). Clear differences were observed between the two noncanonical signals. G6PD6_*medial* with the rice PTS1 (-PTLSKF>) exhibited faint peroxisomal signals, while the one with the maize PTS1 (-PTLSKV>) showed no overlap with the peroxisomal marker and only high cytosolic background (Fig. 7b). Of note, several more members of the *Poaceae* were found to carry a noncanonical PTS1 attached to the conserved ‘PTL’ (Supplementary Fig. S9).

### *Arabidopsis g6pd1 g6pd4 g6pd5 g6pd6* quadruple mutants are viable

To test for redundancy among the *Arabidopsis* isoforms that may localize in peroxisomes, we first crossed single-mutant plants of *g6pd1* and *g6pd4*, which yielded vital *g6pd1 g6pd4* double-mutants (Meyer T, unpublished) that were then crossed with *g6pd5 g6pd6*. Surprisingly, we found homozygous quadruple mutant plants in the F2 progeny. Prompted by a recent study with a different *g6pd5* allele (from now on referred to as *g6pd5-2*; Ruan et al. 2022), we sequenced across the T-DNA border of the allele used by us (from now on referred to as *g6pd5-1*; Wakao and Benning 2005), which revealed attachment of three unrelated amino acids to the C-terminal PTS1 motif (Supplementary Fig. S11A). As expected from the results presented in this study, the GFP-G6PD5_*C-short*-PTLRTN> version did not label peroxisomes (Supplementary Fig. S11B), leaving mutants of the P2 isoforms for further investigations (Supplementary Fig. S11C).

### Seeds of *g6pd2 g6pd3* double mutants show aberrant phenotypes

**Figure 8.**
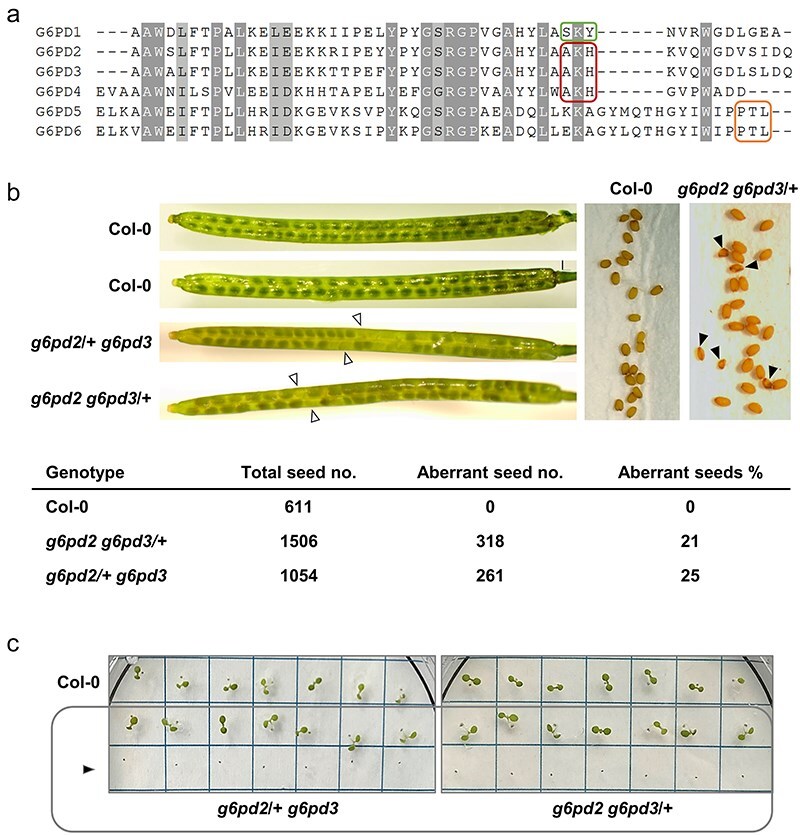
Defective seed development of *g6pd2 g6pd3*/+ and *g6pd2*/+ *g6pd3* double-mutant plants. (a) Alignment of the C-terminal amino acid sequences of the six *Arabidopsis* G6PD isoforms. The internal PTS1 motif of P1 isoform G6PD1 is outlined in green, the corresponding tripeptides of the P2 isoforms G6PD2 and G6PD3, including catalytically inactive P0 isoform G6PD4, are outlined in red, and the exposed weak PTS1 of cytosolic isoforms G6PD5 and G6PD6 is outlined in orange. (b) Analysis of siliques and seeds from *g6pd2 g6pd3*/+ and *g6pd2*/+ *g6pd3* double-mutant plants compared to *A. thaliana* wild-type (Col-0). Immature siliques of *g6pd2 g6pd3*/+ and *g6pd2*/+ *g6pd3* plants contained 21% and 25% whitish seeds, respectively (left, white triangles), with shrunken appearance after desiccation (right, black triangles). (c) Note that the shrunken seeds did not germinate (black triangle) when placed below ‘normal’ seeds of each genotype on the same agar plate.

Concerning potential redundancy among the plastidial G6PD enzymes, it is intriguing that *Arabidopsis* P2 isoforms G6PD2 and G6PD3 carry the tripeptide -AKH-, which aligns with the internal -SKY- motif of P1 isoform G6PD1 (Fig. 8a) and is considered a potential PTS1 signal (Deng et al. 2024). Taking into account that also the catalytically inactive P0 isoform G6PD4 contains this tripeptide (mediating peroxisomal localization of G6PD1; Meyer et al. 2011), there may be further redundancy among the P2 isoforms of heterotrophic tissues. Of note, reciprocal crossing of viable *g6pd2* and *g6pd3* single mutant plants did not result in double homozygous offspring, and neither did repeated selfing of the hemizygous siblings. Numerically, this showed as genotypic frequency of 44% wild-typical and 56% heterozygous offspring from *g6pd2 g6pd3*/+ (*n* = 64), and 25% wild-typical and 75% heterozygous offspring from *g6pd2*/+ *g6pd3* (*n* = 59) mother plants. To find an answer for this aberrant segregation, we inspected siliques of the hemizygous double-mutant combinations. Of note, immature siliques contained whitish seeds, and after ripening, shrunken seeds with a percentage of 21% for *g6pd2 g6pd3/+* and 25% for *g6pd2/+ g6pd3*, respectively (Fig. 8b). Further experiments showed that the shrunken seeds were not able to germinate (Fig. 8c), suggesting that homozygous double mutants of the P2 isoforms are lethal.

## Discussion

The clear peroxisomal localization of reporter fusions with the last 50 amino acids of the G6PD5.1 and G6PD6 (*C-short*) constructs was quite astonishing, since -PTL> seemed to be a rather weak PTS1 motif. In fact, they were able to compete with our soluble peroxisome marker, a *C-short* version of the dual plastid/peroxisomal PGL3 carrying its native canonical PTS1 motif -SKL> (Chowdhary et al. 2012). However, we did not spot the G6PD5 or G6PD6 *full-length* proteins inside peroxisomes, leading to the assumption that there have to be more prerequisites for successful peroxisomal import besides a valid targeting motif, which would be in line with our findings concerning the regulated peroxisomal targeting of 3^rd^ OPPP enzyme PGD2 (Doering et al. 2024).

To overcome the potential interference of N-terminal reporter fusions, we devised and cloned active *medial* constructs. According to a G6PD5 model based on the human G6PD 3D structure, two potential insertion sites for GFP were identified that should not interfere with multimerization, but only one of the two *medial* constructs (GFP inserted between A^139^ and E^140^) displayed high catalytic activity upon transfection of *g6pd5-1 g6pd6-2* mutant protoplast (as in Linnenbrügger et al. 2022). Interestingly, its activity was even higher compared to the N-terminal GFP-G6PD5 fusion, indicating suitability of this strategy for further experiments. A G6PD6_*medial* construct with analogous GFP insertion showed similar activity compared to the N-terminal GFP-G6PD6 fusion, which may be connected to differences between the two cytosolic isoforms concerning kinetic parameters (Wakao and Benning 2005) and enzyme activation by phosphorylation (Dal Santo et al. 2012). Furthermore, GFP exposed on the protein surface could interfere with the binding of interaction partners. Additionally, one has to take into account that our activity measurements are only rough estimates, since the fusion protein amounts are based on band intensities of anti-GFP immunoblots, questioning full comparability of different independent experiments. But since G6PD5.1_*medial* was always used as control and taken as 100%, we believe that this approach is valid to estimate the relative catalytic activity of different constructs in the same background (*g6pd5-1 g6pd6-2* double-mutant protoplasts) without going through the time-consuming procedure of establishing stable plant lines.

We next analysed different deletions and alterations of the PTS1 motif, which showed that peroxisomal import of G6PD5.1 and G6PD6 entirely depends on the -PTL> tripeptide. Yet, there seem to be other regulatory mechanisms that were overruled by a motif change from -PTL> to -SKL> or -SRL>, since *full-length* constructs were not imported in a detectable manner, unless the motif was changed to a canonical one. Additionally, our findings concerning localization of *E. coli* ZWF showed that *Arabidopsis* G6PD5.1 and G6PD6 are destined for peroxisomal import, since *full-length* ZWF was not spotted in peroxisomes, even with -SRL> attached. Considering earlier publications on (un)favourable PTS1 upstream contexts (Chowdhary et al. 2012, Deng et al. 2022), a tryptophane at position −6 is highly disadvantageous (Supplementary Fig. S12), but since found in all homologs of the species analysed—even for *E. coli* ZWF—this residue seems to be important for G6PD function. Furthermore, the highly conserved proline at position −4 seems advantageous. In fact, Skoulding et al. (2015) reported that an exchange to asparagine at this position abolished peroxisomal targeting, providing an explanation for why ZWF-SRL> did not enter peroxisomes. Together with the results obtained for the cytosolic PGI (cPGI) variants, our data support that the upstream region in front of the PTS1 motif can be crucial for peroxisomal import (Chowdhary et al. 2012, Deng et al. 2022). Furthermore, the localization studies with cPGI showed that even though -PTL> was able to compete with -SKL> in the G6PD_*C-short* context, this was not the case for cPGI*_C-short*-PTL>, but the -SRL> version. Thus, there is a preference at the motif level as well—possibly by differences in PEX5-binding affinity (Skoulding et al. 2015). Also, cPGI natively ends -PQM>, which, according to Deng et al. (2024), was a functional PTS1 when fused to an optimized upstream auxiliary peptide (Deng et al. 2022). But there seem to be more parameters that influence peroxisomal import (also mentioned in Deng et al. 2024), which our previous analyses of *Arabidopsis* OPPP enzymes G6PD1/G6PD4, PGL3/PGL5, and PGD2 have shown already—and we here show again for G6PD5 and G6PD6.

In Doering et al. (2024), we elucidated the molecular basis for the preferred peroxisomal import of PGD2 monomers and proceeded to investigate the relevance of the G6PD dimerization status. In a first attempt, we tested this by means of localization studies, coexpressing *full-length* versions with either -PTL> or canonical -SKL>. Of course, both versions may enter peroxisomes on their own and would then appear as overlapping in the matrix, but since the localization pattern of the -PTL> constructs changed that drastically, we assumed the results could also reflect dimeric import. This notion was enforced by the expression of a reporter-free G6PD6-SKL> version in combination with a *medial* GFP fusion without functional PTS1 motif, which led to peroxisomal localization as well, confirming the possibility of dimeric import for G6PD.

Phylogenetic analyses indicate that there must be a physiological reason for cytosolic G6PD enzymes ending -PTL>, which was found especially well conserved among the angiosperms (last 15 amino acids). Interestingly, within the grasses two homologous isoforms end either with -PTLA> or with a noncanonical PTS1 motif (following the definition of Chowdhary et al. 2012) that is added to the well-conserved ‘PTL’. We investigated the localization of rice and maize *C-short* fusions as representatives of the *Poaceae*, confirming potential for peroxisomal import of isoforms ending with a double PTS1 motif. In contrast, an *Arabidopsis C-short* construct ending -PTLA> led to cytosolic distribution. But again, for the G6PD6 medial *full-length* versions ending the same, peroxisomal localization was only partial for -PTLSKF> (rice), while -PTLSKV> (maize) showed none, and -PTLSKL> (ending with a canonical PTS1) complete overlap with the peroxisomal marker. These results suggest that the *Poaceae* developed (and kept) two cytosolic G6PD homologs, one for remaining in the cytosol and one with potential for peroxisomal import, like in the other Angiosperms that retained only G6PD isoforms with the ‘weak’ PTS1 motif -PTL>. Possibly, there are regulatory means that lead to a more effective import of those isoforms when G6PDH activity is needed inside peroxisomes. To this end, we tested for effects of annotated phosphorylation sites in the N-terminus of G6PD5/6 (S12, T13, and S18) using phospho-mimetic and de-mimetic changes as in Doering et al. (2024)—alone and in combination, but none led to a change in the localization pattern. Hence, we concluded that G6PDH activity may be beneficial in the cytosol and mandatory under specific conditions inside peroxisomes, wherefore plants evolved different ways to enable peroxisomal import of G6PD isoforms that may be sufficient without disrupting the cytosolic OPPP.

To test this assumption genetically, we first obtained *g6pd1 g6pd4* double-mutant plants that were then crossed with the *g6pd5-1 g6pd6-2* double-mutant line (Linnenbrügger et al. 2022). Interestingly, *g6pd1 g6pd4 g6pd5 g6pd6* quadruple mutants were found among the F2 progeny, and analyses of the *g6pd5-1* allele (originally used by Wakao and Benning 2005) showed that if there should be residual expression, the C-terminally altered protein is likely not imported. Hence, either G6PDH activity is dispensable inside peroxisomes during fertilization, or G6PD5-PTLRTN> enters peroxisomes by another mechanism. In fact, there are several other NADPH-regenerating pathways in the cytosol, e.g. cytosolic ICDH (Mhamdi et al. 2010), but concerning stages of mainly sugar metabolization in peroxisomes, peroxisomal ICDH could not compensate (Hölscher et al. 2016, Leterrier et al. 2016). Alternatively, the plastidial P2 isoforms are dually targeted to plastids and peroxisomes as well, contributing in two ways: first during pollen and ovule maturation plus pollen tube growth towards ovules, and second during embryo development and seed maturation (Niewiadomski et al. 2005, Andriotis and Smith 2019). Of note, *Arabidopsis* mutants that are deficient in peroxisomal biogenesis factor PEX16, which is implicated in peroxisomal membrane protein targeting and formation of nascent peroxisomes from the ER (Burkhart et al. 2019), were not vital and showed a shrunken seed phenotype (*sse1*; Lin et al. 1999, 2004), and also maize mutants lacking plastidial 6PGDH activity (Spielbauer et al. 2013). But the cytosolic G6PD isoenzymes also contribute during these developmental phases, either by providing NADPH to reactive burst oxidase homologs (Rbohs) at the plasma membrane during apoplastic bursts (Yang et al. 2019), or by supporting e.g. lipid biosynthesis at the cytosolic face of the ER (Linnenbrügger et al. 2022, Ruan et al. 2022).

Our *Arabidopsis* mutant approach to answer the question of extended redundancy among the remaining P2 isoforms reached its limits when no *g6pd2 g6pd3* double homozygous progeny could be found. Defects in the plastidial OPPP part are usually embryo-defective, like *pgl3* (EMB2024; Tzafrir et al. 2004, Xiong et al. 2009, Bussell et al. 2013), or lead to whitish seeds that are unable to germinate after ripening (this work). Similar observations were made for *pgd1 pgd3* double-mutant combinations that neither produced homozygous offspring and—besides whitish seeds—also left empty places in siliques (Andriotis and Smith 2019). Yet, mutant seeds of ribulose-phosphate 3-epimerase (At5g61410, EMB2728) that interconverts Ru5P and Xu5P in plastids still germinated, but *rpe* seedlings remained white and died on soil (unpublished observation of our group). In contrast, a solely peroxisomal defect of *PGD2* (Doering et al. 2024) led to full siliques, but all seedlings exhibited a heterozygous or wild-typical genotype (Hölscher et al. 2016). Dual peroxisomal ER/plastidial defects showed combined phenotypes, like heterozygous plants of glucose 6-phosphate/phosphate transporter GPT1 that produced abnormal pollen and siliques with unfertilized ovules (empty places), but also aborted seeds of different stages (Niewiadomski et al. 2005, Baune et al. 2020, Zhang et al. 2021).

Unambiguous analyses are further complicated by compensatory up- or downregulation of other G6PD isoforms—already in *g6pd1* single-mutant plants, as recently shown by Landi et al. (2024). This led to a stress-priming effect that was most beneficial under cold temperatures. In the *g6pd5-2 g6pd6* double mutant, especially *G6PD2* expression was upregulated (Yang et al. 2019). Therefore, pinpointing the exact role and contribution of G6PD isoforms in different subcellular compartments is challenging. A next task will be to test how siliques of the P2 mutant combinations with or without P1 isoform G6PD1 perform during fertilization and seed development.

**Figure 9.**
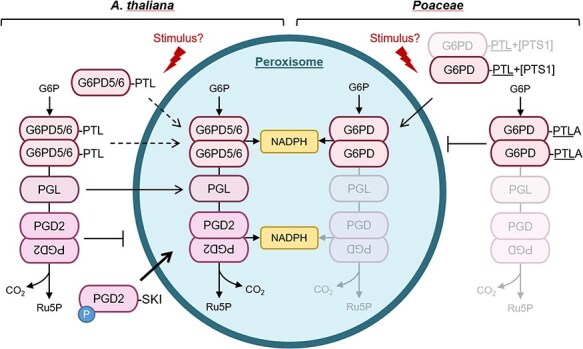
Summary scheme of cytosolic G6PD isoform contribution to OPPP activity in peroxisomes. Based on the results obtained in this and our previous studies on OPPP reactions in plant cells, we propose that members of the Angiosperms developped two different ways to ensure dual cytosolic and peroxisomal G6PDH activity. Left, the situation in *A. thaliana* (as an example for Angiosperms without the *Poaceae*), where both G6PD5 and G6PD6 possess a ‘weak’ PTS1 motif (-PTL>) and get imported in small quantities (dashed arrows), likely also as homo- or heterodimers so that a basic level of activity is guaranteed inside peroxisomes. Right, the situation in the *Poaceae*, where two different isoforms are present. One solely cytosolic (-PTLA>), and one with a stronger PTS1 motif attached to the conserved ‘PTL’ (-PTL+[PTS1]), allowing for a basal level of G6PD import into peroxisomes (possibly also as dimers, shaded). Upon an unknown stimulus (red flash sign), stronger import of these isoforms may be achieved. Alternatively, G6PD1 import upon cellular redox changes (Meyer et al. 2011) may be sufficient. For the second OPPP step, there is extensive redundancy for the peroxisomal part, since all five isoforms were able to enter peroxisomes - some via piggybacking (Lansing et al. 2020), but PGL3 seems to be the most important isoform in case of high metabolic flux requirements. PGD2 is present in peroxisomes at low levels, but likely not imported as dimer. Upon N-terminal phosphorylation mimicry (T6E or T6D), monomeric import was drastically enhanced (bold arrow; Doering et al. 2024), likely to promote NADPH provision by the OPPP in peroxisomes. For the last two enzymes of the *Poaceae*, the situation may be very similar but this was not tested experimentally in our study.

A final summary scheme (Fig. 9) illustrates our current view of OPPP completion in peroxisomes of *A. thaliana* versus the *Poaceae*. Under normal conditions, there is probably only basal import of G6PD5.1 and G6PD6 into the matrix, either in monomeric or dimeric form (Fig. 9, left). In case of an unknown stimulus, the two isoforms may be more efficiently imported. In rice and maize (Fig. 9, right), a second set of G6PD isoforms without functional PTS1 (-PTLA>) remains in the cytosol, while isoforms with a double PTS1 motif can enter peroxisomes. Whether the latter may be increased by a stimulus is unknown. At least N-terminal phosphorylation may efficiently direct the *Arabidopsis* PGD isoforms to organelles: PGD1 and PGD3 to plastids (not shown) and PGD2 to peroxisomes (Doering et al. 2024). As a consequence, 6-phosphogluconate (6PG) levels may rise in the cytosol, possibly supported by higher activity of phosphorylated G6PD6 (Dal Santo et al. 2012). We recently showed for tobacco that 6PG backup results in elevated fructose 2,6-bisphosphate levels and partial sugar retention in a stress-linked manner (Scharte et al. 2023). Moreover, enhanced availability of soluble sugars promoted the expression of cytosolic *G6PD* isoforms in potatoes (Hauschild and von Schaewen 2003) and also in *Arabidopsis* (eFP browser light series; Winter et al. 2007). Furthermore, alternative splicing in plants is known to be mainly stress-related (Martín et al. 2021), which led to N-terminally extended *Arabidopsis* G6PD5 proteoforms that were inserted into the outer face of the ER, and whose abundance differed upon germination in the presence versus absence of sucrose (Linnenbrügger et al. 2022). All these plant-specific features may guarantee that some G6PDH activity is retained in the cytosol of most Angiosperms (Fig. 6), if the cytosolic G6PD isoforms ending with the same ‘weak’ PTS1 motif should both be directed to peroxisomes by an unknown trigger. To elucidate this remains a task of prospective research.

## Materials and Methods

### Bioinformatics

Sequence information on *Arabidopsis* genes and proteins was retrieved from the plant membrane protein database ARAMEMNON (https://aramemnon.botanik.uni-koeln.de/) of the Institute of Plant Science, University of Cologne, Germany. Sequence alignments were performed with Clustal Omega (https://www.ebi.ac.uk/jdispatcher/msa/clustalo) provided by the EMBL-EBI (European Molecular Biology Laboratory—European Bioinformatic Institute, Heidelberg, Germany). 3D models were acquired from the protein structure database AlphaFold (https://alphafold.ebi.ac.uk/) (via EMBL-EBI) and modified with Protean 3D (DNASTAR, Inc.). Further information was retrieved from The Arabidopsis Information Resource (TAIR, https://www.arabidopsis.org/), which is supported by Phoenix Bioinformatics. Information about post-translational modification sites was collected from PhosPhAt (https://phosphat.uni-hohenheim.de/) and the Plant PTM Viewer (https://www.psb.ugent.be/webtools/ptm-viewer/).

### *Arabidopsis* mutants

As described earlier (Linnenbrügger et al. 2022), seeds of the *g6pd5-1 g6pd6-2* double mutant were kindly provided by Claudia Jonak (Vienna, Austria) after crossing (SALK_045083 × GK_142G07). The *g6pd1 g6pd4 g6pd5-1 g6pd6-2* quadruple mutant was obtained by first crossing single-mutant plants of *g6pd1* (GK_864A05) and *g6pd4* (GK_904H12), followed by crossing confirmed double-mutant plants. The hemizygous *g6pd2 g6pd3*/+ and *g6pd2*/+ *g6pd3* lines of the P2 isoforms were generated by crossing single-mutant plants of *g6pd2* (GK_319B08.02) and *g6pd3* (SALK_139479). For further analyses, hemizygous plants of the F2 were allowed to repeatedly self-pollinate (F3, F4, etc.).

### Plant growth

*Arabidopsis* wild-type (*A. thaliana* var. Columbia) and *g6pd5-1 g6pd6-2* double-mutant plants for protoplast isolation were grown in tissue culture as described earlier (Linnenbrügger et al. 2022). Plants for genotyping were first surface sterilized and grown under sterile conditions, but after 1–2 weeks on germination plates [½ Murashige and Skoog salts with vitamins, pH 5.7, 0.8% (w/v) agar and 1% (w/v) sucrose] they were to soil (or the same medium in Magenta™ boxes, SIGMA) and further grown under short-day regime (8-h light, 22°C/16-h darkness, 20°C).

### Isolation of genomic DNA and characterization of mutant lines

Genomic DNA was isolated from leaf material as in Hölscher et al. (2016). PCR reactions were performed with either two gene-specific primers or one gene- and one T-DNA-specific primer (listed in Supplementary Table S1). For analytical purposes, Taq DNA Polymerase (Biozym Scientific GmbH, Germany) was used. DNA fragments were analysed by gel electrophoresis [1% agarose (w/v), Carl Roth, Germany].

### Cloning of fluorescent reporter fusions

Fluorescent reporter constructs were generated as described earlier (Hölscher et al. 2016, Baune et al. 2020, Lansing et al. 2020, Linnenbrügger et al. 2022, Doering et al. 2024). Open reading frames were amplified from either seedling cDNA or existing plasmid constructs using S7 Fusion High-Fidelity DNA Polymerase (Biozym Scientific GmbH, Germany) with proof-reading activity, inserted into the plant expression vectors via compatible restriction sites, and verified by sequencing. For oligonucleotide primers used in this study, see Supplementary Table S1, with constructs indicated.

### Site-directed mutagenesis

Single amino acid changes or premature stop codons were introduced at the nucleotide level using a PCR-based site-directed mutagenesis protocol originally from Stratagene (now QuikChange Site-Directed Mutagenesis Kit, Agilent Technologies) using S7 Fusion High-Fidelity DNA Polymerase (Biozym Scientific GmbH, Germany). For primer combinations, see Supplementary Table S1. All base changes were verified by sequencing.

### Cloning of double cassette constructs

First, the reporter-less construct was cloned by the strategy described in Linnenbrügger et al. (2022), using primers 1202 and 1203 (Supplementary Table S1). Second, G6PD6_*medial* was mutagenized using primers 1903 and 1904 to delete the last leucine, resulting in G6PD6_*medial*-∆L. Then, the reporter-less expression cassette was inserted via NotI and SdaI restriction sites into G6PD6_*medial*-∆L and confirmed by restriction digests. All constructs were verified by sequencing.

### Protoplast transfection

Transfection of *Arabidopsis* protoplasts was conducted by a polyethylene glycol (PEG) method (Damm et al. 1989). Leaves of 4- to 5-week-old sterile sugar-adapted plants were harvested into a Petri dish containing 0.45 M mannitol solution and incubated for 1 h. The osmotic agent was then replaced by an enzyme solution containing 0.45 M mannitol, 10 mM CaCl_2_, 1% (w/v) cellulase, and 0.25% (w/v) macerozyme (Onozuka™ grade). The leaf parts were incubated in a Parafilm™-sealed Petri dish overnight (∼16–20 h) in the dark and gently swivelled at 23°C. Then, the enzyme solution was diluted one-to-one with W5 medium (145 mM NaCl, 125 mM CaCl_2_, 5 mM KCl, 5 mM glucose, pH 5–6) and filtered through a sieve combination (stainless steel 180 µm, 63 µm), a washing step included. The filtered protoplast solution was transferred to a glass centrifugation tube and spun at 60 g and 15°C for 5 min. The supernatant was discarded and the protoplast pellet was taken up in fresh W5 medium, followed by 1 h incubation on ice. After a second centrifugation step, the supernatant was discarded and the protoplasts were adjusted to a concentration of 1.67 · 10^6^ cells·ml^−1^ with ice-cold MaMg solution (0.45 M mannitol, 15 mM MgCl_2_, 0.1% MES, pH 5.7). Approximately 500 000 protoplasts (300 µl of the protoplast solution) were transfected with premixed plasmid constructs (concentration 0.5–1 µg·µl^−1^) by slowly dripping 500 µl of PEG4000 onto protoplasts mixed with the DNA. Generally, 15 µg of plasmid DNA was used for *full-length* constructs, 10 µg for *short*-version constructs, 7 µg for PEX16-OFP, and 5 µg for OFP-PGL3_*C-short*. After 30 min of incubation at room temperature, the protoplasts were diluted stepwise with a total of 7 ml of W5 medium and harvested by centrifugation. At last, protoplast pellets were taken up in 1.5 ml of Gamborg B5 medium with vitamins (Duchefa Biochemie, The Netherlands) plus glucose and hormones (3.17 g·l^−1^ Gamborg B5, 0.45 M glucose, pH 5.7, supplemented with 1 mg·L^−1^ 2,4-dichlorophenoxyacetic acid, and 0.15 mg·L^−1^ 6-benzylaminopurine) and incubated in the dark at room temperature for 24–48 h.

### Confocal laser scanning microscopy

Localization studies were performed with a confocal laser-scanning microscope (Leica TCS SP5 or Leica TCS SP8). The following settings were used for excitation/emission wavelengths: 488/490–520 nm for GFP and 561/590–620 nm for OFP (Lansing et al. 2020).

### G6PDH activity

Enzymatic measurements were conducted essentially as described in Linnenbrügger et al. (2022). *Arabidopsis g6pd5 g6pd6* mutant mesophyll protoplasts were transfected with GFP fusion constructs that were used for localization studies as well. Protoplasts incubated solely with TE buffer served as a negative control. In contrast to localization studies, 2–3 Mio protoplasts were transfected separately with 30 µg of plasmid DNA per 1 Mio each, incubated in 3 ml of Gamborg B5 medium with glucose and hormones, and pooled ∼24 h after transfection. Cells were harvested by two centrifugation steps (60 g, 5 min; 100 g, 3 min) in a 2-ml reaction vial. The supernatant was discarded, initially until only about 500 µl of the suspension remained, then completely. The protoplast pellet was resuspended in 100–150 µl of extraction buffer [100 mM HEPES, pH 7.5, 2 mM Na_2_S_2_O_5_, 5 µl protease inhibitor cocktail (for use with plant extracts, Sigma), and 0.02% Triton X-100]. Cells were ruptured by means of mechanical shear forces (pipetting up and down with a 200-µl tip). For SDS-PAGE analyses, 37.5 µl from each sample was transferred into a fresh 1.5-ml reaction vial and, if necessary, shock-frozen in liquid nitrogen and stored at −80°C. For G6PDH activity measurements, 20–40 µl of each sample was mixed with assay buffer, containing 100 mM TRIS-maleate, pH 8, 0.2 mM NADP^+^, and 2 mM G6P (to start the reaction). Conversion of NADP^+^ to NADPH^+^ H^+^ was recorded photometrically at 340 nm (at room temperature) every 12–15 s using different aliquots. The slope over a linear range was taken as a measure of activity.

### Immunoblot analyses

Immunoblot analyses were performed as described previously in Linnenbrügger et al. (2022).

### Phenotypic silique analysis

Immature siliques were immersed in 8 M sodium hydroxide (NaOH) for 2 days. The solution was eventually renewed after 1 day. Ripe siliques were harvested individually and opened for documentation of fertilization and seed set. Photographs were taken with a camera (Canon 9000 Mark II) attached to a binocular (Olympus SZ61 + DP25).

## Supplementary Material

pcaf012_Supp

## Data Availability

Biological materials and data not shown in supplementary data can be made available on request.
