# Supplementary material for: Evidence for peroxisomal redundancy among the glucose-6-phosphate dehydrogenase isoforms of Arabidopsis thaliana
Source: Plant Cell Physiol. 2025 Jan 18;66(5):722–37. doi: 10.1093/pcp/pcaf012 (PMC12125578; doi:10.1093/pcp/pcaf012)
Supplement: pcaf012_Supp [file pcaf012_supp.zip › suppl_data/pcp-2024-e-00257-File011.docx]

**Table S1.** Oligonucleotides used in this study (s, sense; as, antisense, WT, wild type, SDM, site directed mutagenesis).

| **Lab no.** | **Name** | **Sequence** | **Construct/Application** |
| --- | --- | --- | --- |
| ***G6PD1*** |  |  |  |
| 300 | GABI_864A05 s | CAGTGCTCCGATTCTCAAATC | Genotyping WT/T-DNA |
| 301 | GABI_864A05 as | AACAACCTCCTCTCTCCTTCG | Genotyping WT |
| ***G6PD4*** |  |  |  |
| 28 | SALK_131208 as | CGTGGTGCCTGAGCGTTATCT | Genotyping WT |
| 29 | SALK_131208 s | GAGGAGGCTCGAATCTCTGCC | Genotyping WT/T-DNA |
| ***G6PD2*** |  |  |  |
| 873 | GK319B08 s | GGAGGAAGATCAAATTTTTAGGTG | Genotyping WT/T-DNA |
| 874 | GK319B08 as | ATTCCACAAGTGCGAAGT | Genotyping WT |
| ***G6PD3*** |  |  |  |
| 298 | SALK_139479 as | TGGTAGGTGTAAGGTGATATGCTG | Genotyping WT |
| 299 | SALK_139479 s | TTGCATTGTCGATGAAGAGTG | Genotyping WT/T-DNA |
| ***G6PD5*** |  |  |  |
| 598 |  | GAAGTTTTTGGCTTTGCTGCG | Genotyping WT/T-DNA |
| 599 |  | GAAGAGTTTTTATTCAGCAAGCAAGG | Genotyping WT |
| 1531 | G6PD5 s | NNNTCTAGAATGGGTTCTGGTCAATGGC | G6PD5.1_*medial* |
| 1735 | G6PD5 N139 as | NNNCCATGGCAGTCTTTTTAGATATCTCGTGC | G6PD5.1_*medial* A^139^ E^140^ |
| 1736 | G6PD5 C377 s | NNNACTAGTGAAGGATCTTCGAGGAGATTG |  |
| 1737 | G6PD5 N75 as | NNNCCATGGCCTTACTCCTTGCATATCC | G6PD5.1_*medial* K^75^ I^76^ |
| 1738 | G6PD5 C441 s | NNNACTAGTATTACTGATGAGGAGCTTAGAG |  |
| 1714 | G6PD5 C-short s | NNNACTAGTTTCACTCCACTGCTTCACAG | G6PD5.1_*C-short* |
| 1715 | G6PD5.1 as | NNNAGATCTTTACAATGTAGGAGGGATC | G6PD5.1_*C-short,*  G6PD5.1_*medial,*  GFP-G6PD5.1 |
| 1891 | G6PD5.1 SDM SKL s | GCTACATTTGGATCCCTTCTAAATTGTAAAGATCC | SDM G6PD5.1_*medial*-SKL> |
| 1892 | G6PD5.1 SDM SKL as | GGATCTTTACAATTTAGAAGGGATCCAAATGTAGC |  |
| 1901 | G6PD5 SDM ∆L s | CATTTGGATCCCTCCTACATAGTAAAGATCC | SDM GFP-G6PD5.1_*C-short-*∆L |
| 1902 | G6PD5 SDM ∆L as | GGATCTTTACTATGTAGGAGGGATCCAAATG |  |
| 2070 | G6PD5 PTLA as | NNNAGATCTTCATGCCAATGTAGGAGGG | GFP-G6PD5.1_*C-short*  +A> |
| ***G6PD6*** |  |  |  |
| 600 |  | GTTGCCATGGAGAAACCAATATCTC | Genotyping WT/T-DNA |
| 601 |  | CACCTTGATACCGTTGCCCATACG | Genotyping WT |
| 942 | G6PD6 s | NNNACTAGTATGGGATCTGGTCAATGGCAC | GFP/OFP-G6PD6,  G6PD6_*medial* |
| 945 | G6PD6 as | NNNGGATCCTTATAGTGTAGGAGGAATCCAGATATAGC | GFP/OFP-G6PD6,  G6PD6_*medial* |
| 1202 | 35S Promotor s | NNNGCGGCCGCTGAGACTTTTCAACAAAGG | Double cassette construct of Fig. 4B |
| 1203 | Nos Terminator as | NNNCCTGCAGGCCGATCTAGTAACATAGATGAC |  |
| 1716 | G6PD6 *C-short* s | NNNACTAGTTTCACGCCGCTACTC | GFP/OFP-G6PD6 _*C-short* |
| 1717 | G6PD6 as | NNNAGATCTTTATAGTGTAGGAGGGATC |  |
| 1816 | G6PD6 s | NNNCTCGAGATGGGATCTGGTCAATG | G6PD6_*medial* T^138^ E^139^ |
| 1817 | G6PD6 N138 as | NNNGGTACCAGTACTATTTTTGGAGATTTCG |  |
| 1818 | G6PD6 C377 s | NNNACTAGTGAAGGGTCTTCTAGAAGACTGTTTTATC |  |
| 1853 | G6PD6 ∆PTL as | NNNAGATCTTTAAGGAATCCAGATATAGC | GFP-G6PD6_*C-short-*∆PTL |
| 1893 | G6PD6 SDM SKL s | CTATATCTGGATCCCTTCTAAACTATAAGGATCCCC | SDM G6PD6_*medial*-SKL>,  OFP-G6PD6-SKL> |
| 1894 | G6PD6 SDM SKL as | GGGGATCCTTATAGTTTAGAAGGGATCCAGATATAG |  |
| 1903 | G6PD6 SDM ∆L s | CTGGATTCCTCCTACATGATAAGGATCCCC | SDM G6PD6_*medial*-∆L |
| 1904 | G6PD6 SDM ∆L as | GGGGATCCTTATCATGTAGGAGGAATCCAG |  |
| 2060 | G6PD6 PTLSKV as | NNNGGATCCTTAGACTTTTGATAGTGTAGGAGGAATCC | G6PD6_medial+SKV> |
| 2061 | G6PD6 PTLSKL as | NNNGGATCCTTAGAGTTTACTTAGTGTAGGAGGAATCC | G6PD6_medial+SKL> |
| 2069 | G6PD6 PTLSKF as | NNNGGATCCTTAGAATTTTGATAGTGTAGGAGGAATCC | G6PD6_medial+SKF> |
| **Rice** |  |  |  |
|  | Os *C-short* s | NNNACTAGTTCAGCAGCACTTCGTTCG | GFP-OsG6PD_*C-short* |
|  | Os *C-short* as | NNNGGATCCTTAGAATTTTGAAAGGGTTGG |  |
| **Maize** |  |  |  |
|  | Zm *C-short* s | NNNACTAGTTCAGCAGCACTTCGTTCG | GFP-ZmG6PD_*C-short* |
|  | Zm *C-short* as | NNNGGATCCTTAAACCTTAGAAAGGGTCG |  |
| ***ZWF*** |  |  |  |
| 1321 | ZWF s | NNNACTAGTATGGCGGTAACGCAAACAG | GFP-ZWF |
| 1322 | ZWF SRL as | NNNGGATCCTTACAGACGGCTCTCAAACTCATTCCAGG | GFP-ZWF+SRL>,  GFP-ZWF_*C-short*+SRL> |
| 1450 | ZWF SKL as | NNNGGATCCTTACAGTTTGCTCTCAAACTCATTCCAGG | GFP-ZWF+SKL> |
| 1890 | ZWF *C-short* s | NNNACTAGTGTGGAAGAAGCCTGGAAATGGGTAGACTCC | GFP-ZWF_*C-short* |
| ***cPGI*** |  |  |  |
| 1991 | cPGI s | NNNACTAGTATGGCGTCATCAACCGCTTTG | GFP-cPGI |
| 1992 | cPGI as | NNNGGATCCTCACATCTGGGGCTCG | GFP-cPGI,  GFP-cPGI_*C-short* |
| 1993 | cPGI *C-short* s | NNNACTAGTTTTGACCAGTGGGGCGTTG | GFP-cPGI_*C-short* |
| 2028 | cPGI SDM PTL s | CAAGTTCCGAGCCCACGTTGTGAGGATCC | SDM GFP-cPGI-PTL>,  GFP-cPGI_*C-short*-PTL> |
| 2029 | cPGI SDM PTL as | GGATCCTCACAACGTGGGCTCGGAACTTG |  |
| 2030 | cPGI SDM SRL s | GACAAGTTCCGAGTCCCGGTTGTGAGGATCC | SDM GFP-cPGI-SRL>,  GFP-cPGI_*C-short*-SRL> |
| 2031 | cPGI SDM SRL as | GGATCCTCACAACCGGGACTCGGAACTTGTC |  |
| **T-DNA** |  |  |  |
| 26 | T-DNA GABI1 | CCCATTTGGACGTGAATGTAGACAC | Genotyping T-DNA G6PD6, G6PD4 |
| 831 | T-DNA GABI2 | ATAATAACGCTGCGGACATCTACATTTT | Genotyping T-DNA G6PD2, G6PD1 |
|  | LBa1 SALK | TGGTTCACGTAGTGGGCCATCG | Genotyping T-DNA G6PD5, G6PD3 |
