# Supplementary material for: Evidence for peroxisomal redundancy among the glucose-6-phosphate dehydrogenase isoforms of Arabidopsis thaliana
Source: Plant Cell Physiol. 2025 Jan 18;66(5):722–37. doi: 10.1093/pcp/pcaf012 (PMC12125578; doi:10.1093/pcp/pcaf012)
Supplement: pcaf012_Supp [file pcaf012_supp.zip › suppl_data/pcp-2024-e-00257-File012.pdf]

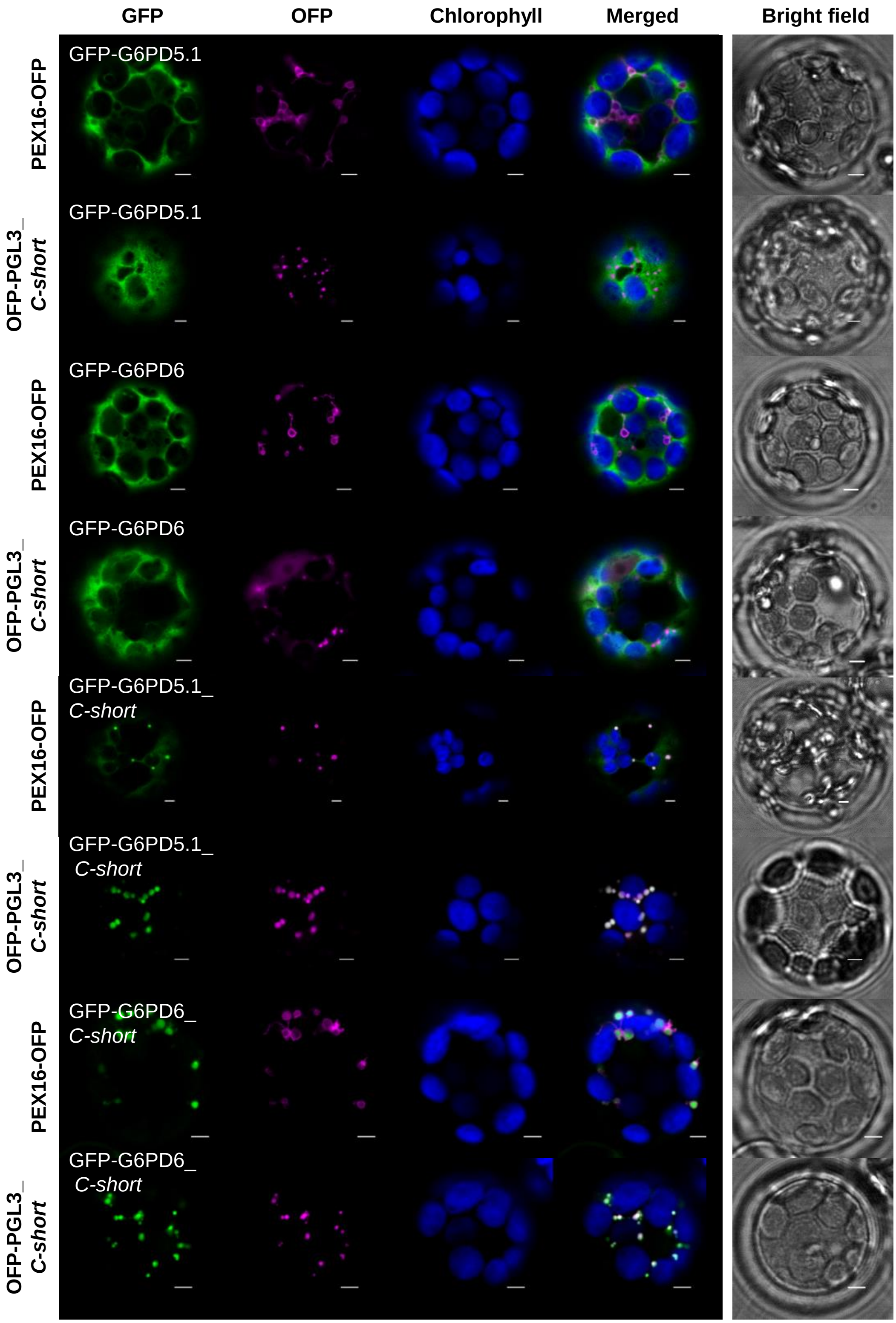

Supplementary Figure S1. Single channel images of Figure 1.

Co-expression of GFP-G6PD5.1 and -G6PD6 reporter fusions (*full-length* and the *C-short* constructs) with two different peroxisomal markers. The images show single optical sections of the three fluorescent channels. Merged, overlay of all channels; Bright field, cellular reference. GFP in green, OFP in magenta, chlorophyll autofluorescence in blue. White signals in Merged indicate co-localisation or very close signals (<200 nm) of GFP and OFP. Scale bars, 3 μm

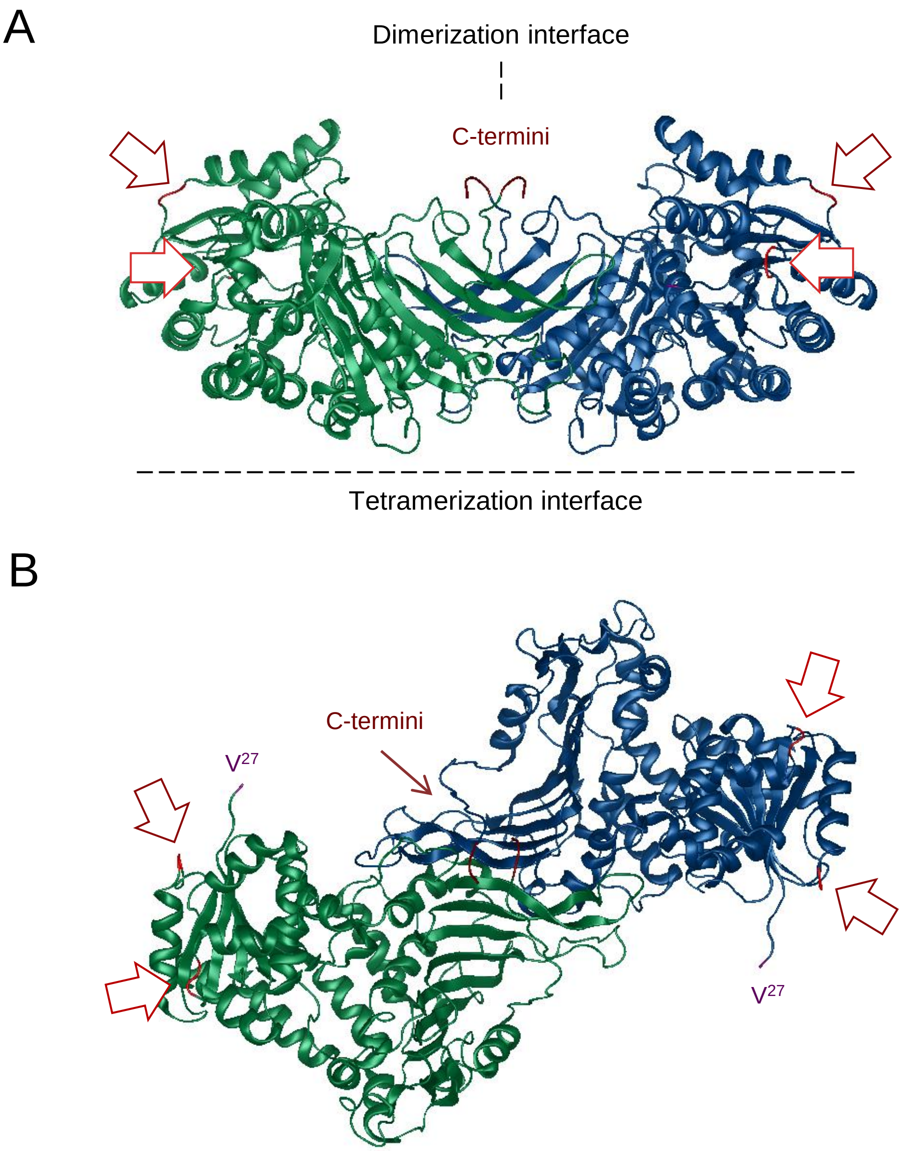

Supplementary Figure S2. 3D structure of human G6PDH dimers.

3D structures of human G6PDH (PDB files obtained from Alphafold and modified with Protean 3D). A) Arrangement of the G6PD5 monomers shown in Figure 2 is based on the human G6PDH tetramer to select suitable positions in outer loop regions for GFP insertion, indicated by white arrows with different red outlines (dark and bright red). B) Rotated dimer of human G6PDH to show the N-termini (purple) and C-terminal dimerization interface from a different angle); the C-termini are highlighted in brown. Note that the 3D models of human G6PDH start with amino acid 27 (valine).

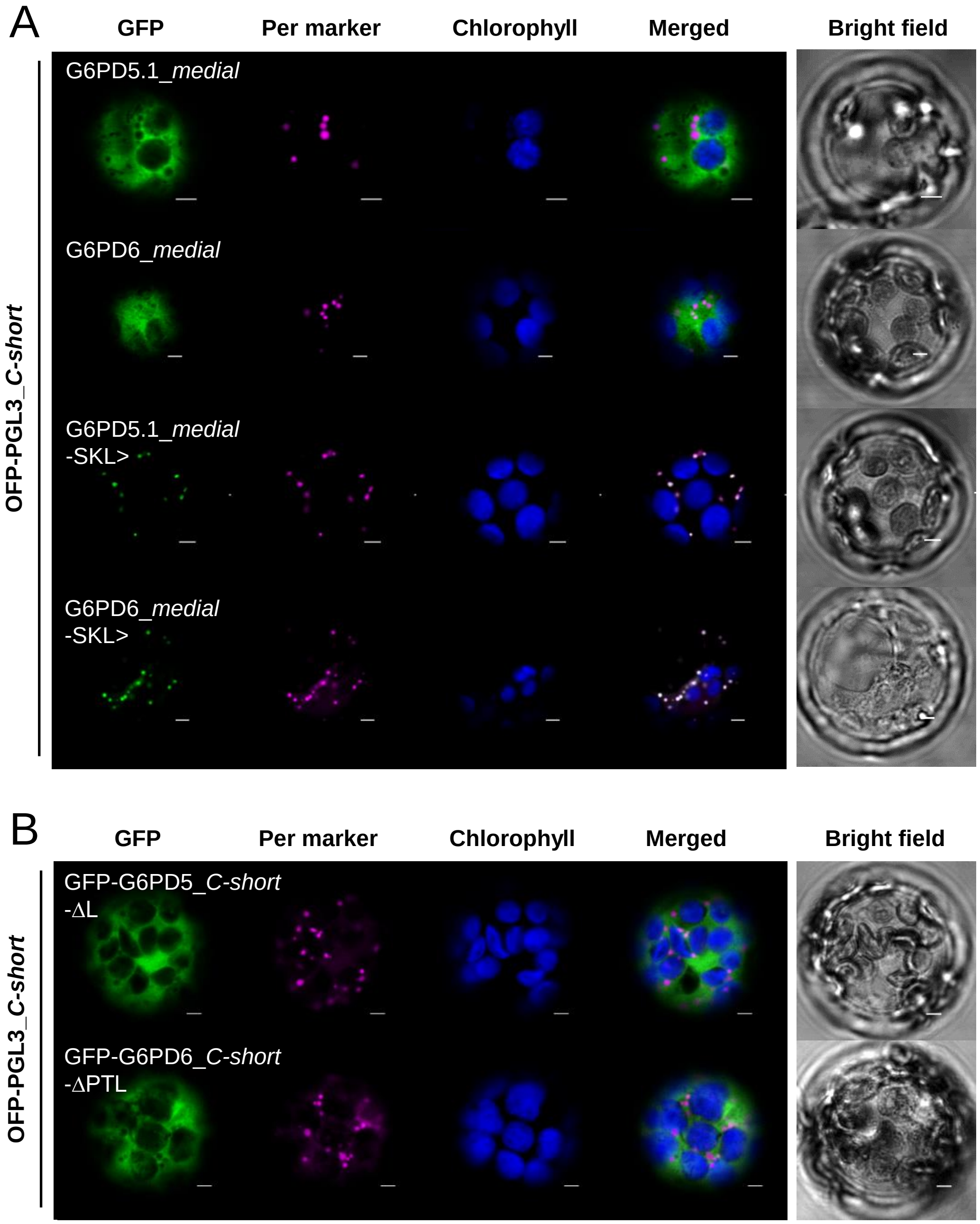

Supplementary Figure S3. Single channel images of Figure 3.

Co-expression analyses with peroxisome (Per) marker OFP-PGL3_*C-short* (ending -SKL>). A) Medial GFP fusions of G6PD5.1 and G6PD6 with wild-typical -PTL> or engineered -SKL>, B) Short variants of the last 50 amino acids of fused to GFP. The images show single optical sections of the three fluorescent channels. Merged, overlay of all channels; Bright field, cellular reference. GFP in green, OFP in magenta, chlorophyll autofluorescence in blue. White signals in Merged indicate co-localisation or very close signals (<200 nm) of GFP and OFP. Scale bars, 3 µm.

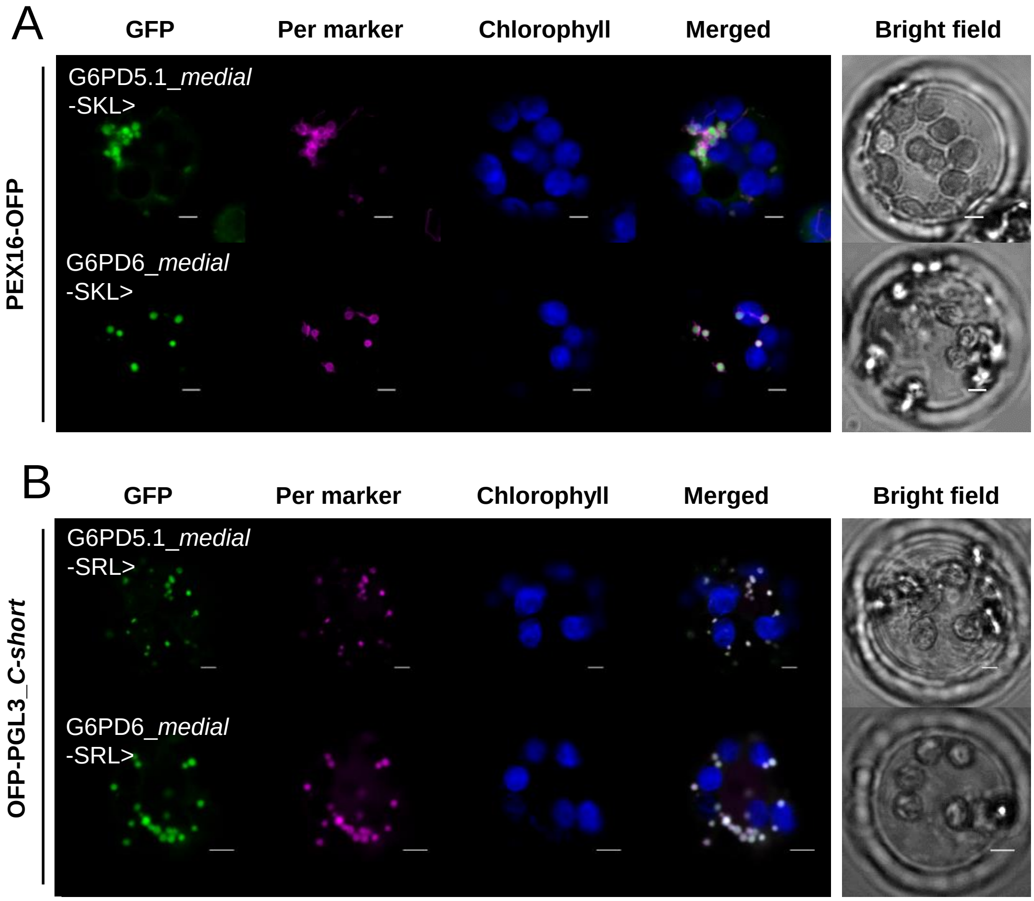

Supplementary Figure S4. Additional localization analyses - supporting Figure 3.

A) Co-expression of the *medial* GFP fusions with engineered PTS1 motif -SKL> and peroxisomal membrane marker PEX16-OFP. B) Co-expression of the *medial* GFP fusions with engineered PTS1 motif -SRL> and peroxisomal marker OFP-PGL3_*C-short* (ending -SKL>). The images show single optical sections of the three fluorescent channels. Merged, overlay of all channels; Bright field, cellular reference. GFP in green, OFP in magenta, chlorophyll autofluorescence in blue. White signals in Merged indicate co-localisation or very close signals (<200 nm) of GFP and OFP. Scale bars, 3 µm.

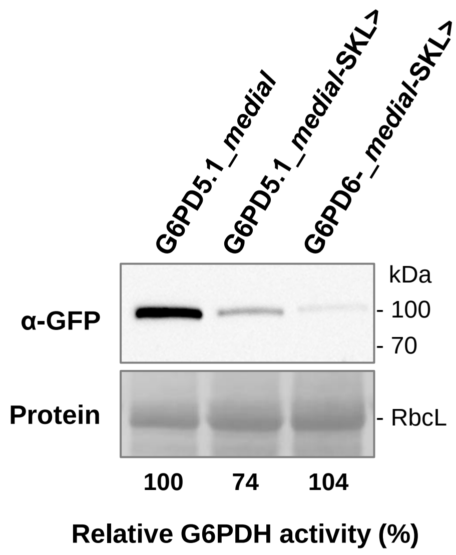

Supplementary Figure S5. The *medial* GFP fusions with engineered PTS1 motif are active.

Immunoblot analysis of protein extracts prepared from *g6pd5-1 g6pd6-2* double mutant protoplasts upon transfection with the indicated *medial* GFP constructs, using anti-GFP antibodies (α-GFP). Protein refers to signals on the Ponceau S-stained blot with RubisCO large subunit (RbcL) as loading reference. G6PDH activity was determined approximately 24 h post-transfection with at least three measurements per extract from three independent experiments. Relative activities were calculated based on corresponding band intensities on the α-GFP immunoblot, with G6PD5.1 _*medial* (ending -PTL>) set to 100%.

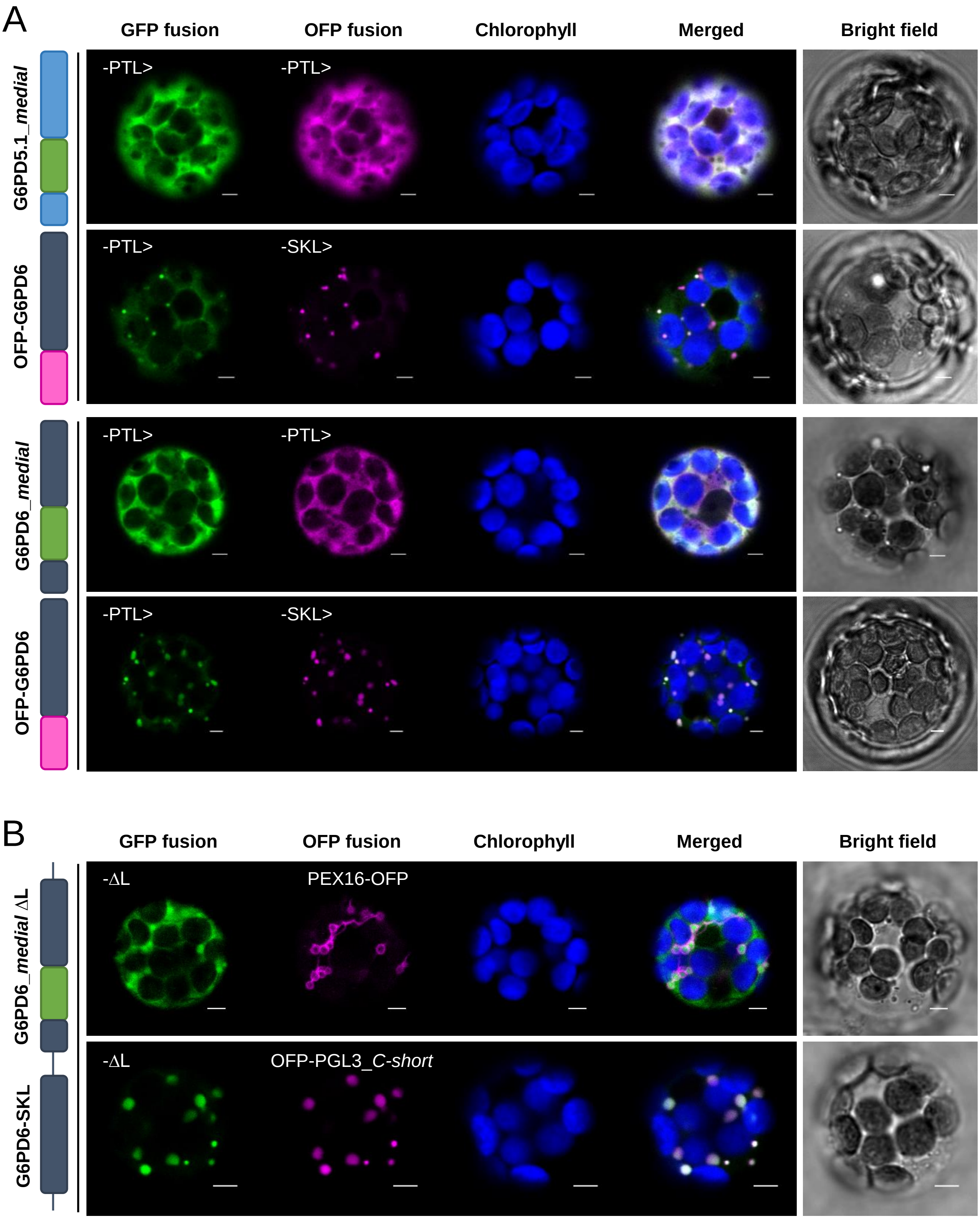

Supplementary Figure S6. Single channel images of Figure 4.

A) Co-expression of the indicated *medial* GFP-fusions with OFP-G6PD6, ending either (-PTL>, upper panels), or with engineered PTS1-motif -SKL> (lower panels). B) Localisation study using a double cassette construct, consisting of G6PD6 ending -SKL> (without reporter) and the G6PD6_*medial* GFP fusion without the last amino acid (-ΔL) on the same plasmid, co-expressed with peroxisomal membrane marker PEX16-OFP (upper panels) or peroxisomal marker OFP-PGL3_*C-short* (ending -SKL>, lower panels). The images show single optical sections of the three fluorescent channels. Merged, overlay of all channels; Bright field, cellular reference. GFP in green, OFP in magenta, chlorophyll autofluorescence in blue. White signals in Merged indicate co-localisation or very close signals (<200 nm) of GFP and OFP. Scale bars, 3 µm.

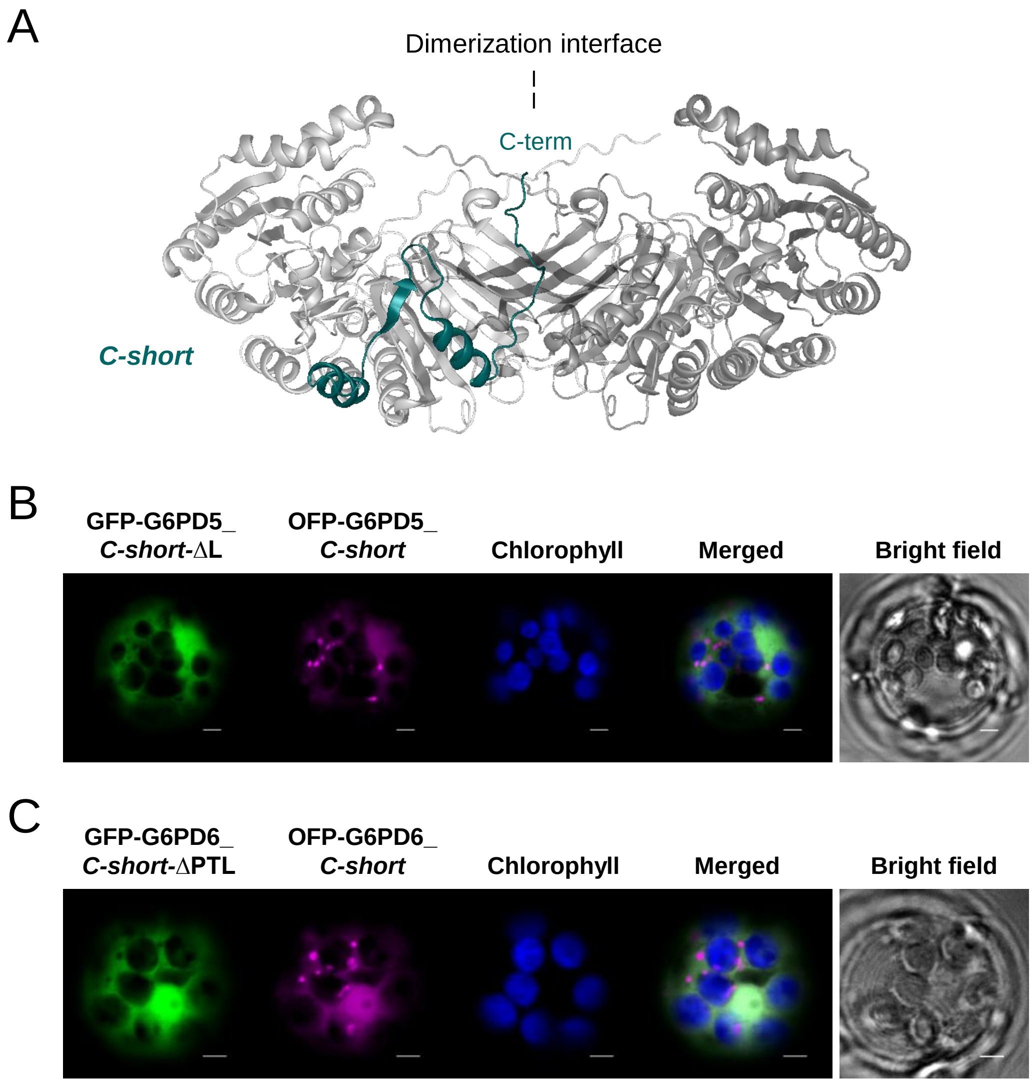

Supplementary Figure S7. The two *C-short* versions do not interact - supporting Figure 4

A) G6PD dimer in grey with the C-terminal *short* part highlighted in turquoise. B) Co-expression of oppositely labelled *C-short* constructs with the GFP variant lacking either only the last amino acid (ΔL) or entire PTS1 motif (ΔPTL). The images show single optical sections of the three fluorescent channels. Merged, overlay of all channels; Bright field, cellular reference. GFP in green, OFP in magenta, chlorophyll autofluorescence in blue. White signals in Merged indicate co-localisation or very close signals (<200 nm) of GFP and OFP. Scale bars, 3 μm.

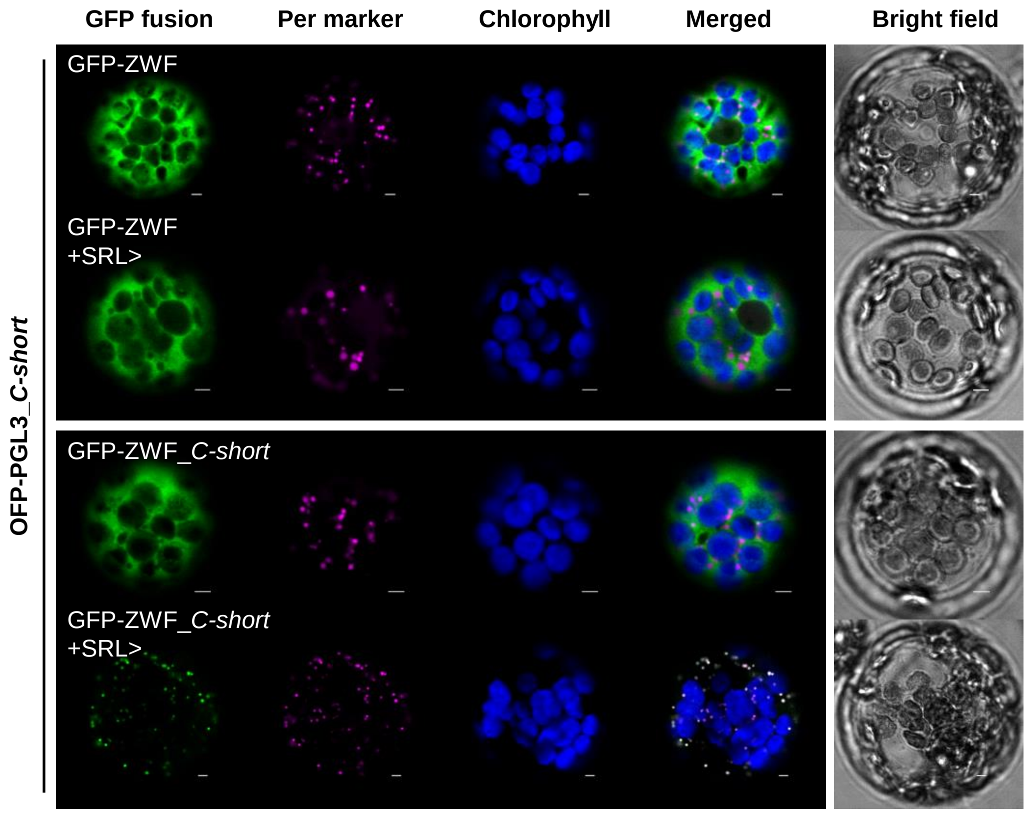

Supplementary Figure S8. Single channel images of Figure 5A.

Co-expression of peroxisomal marker OFP-PGL3_*C-short* (ending –SKL>) with different variants of the G6PDH of *Escherichia coli* (ZWF, '*Zwischenferment*') fused to GFP, either *as full-length* version (top), or the last 50 amino acids (*C-short*), ending either wild-typically or with added PTS1 motif -SRL>. The images show single optical sections of the three fluorescent channels. Merged, overlay of all channels; Bright field, cellular reference. GFP in green, OFP in magenta, chlorophyll autofluorescence in blue. White signals in Merged indicate co-localisation or very close signals (<200 nm) of GFP and OFP. Scale bars, 3 µm.

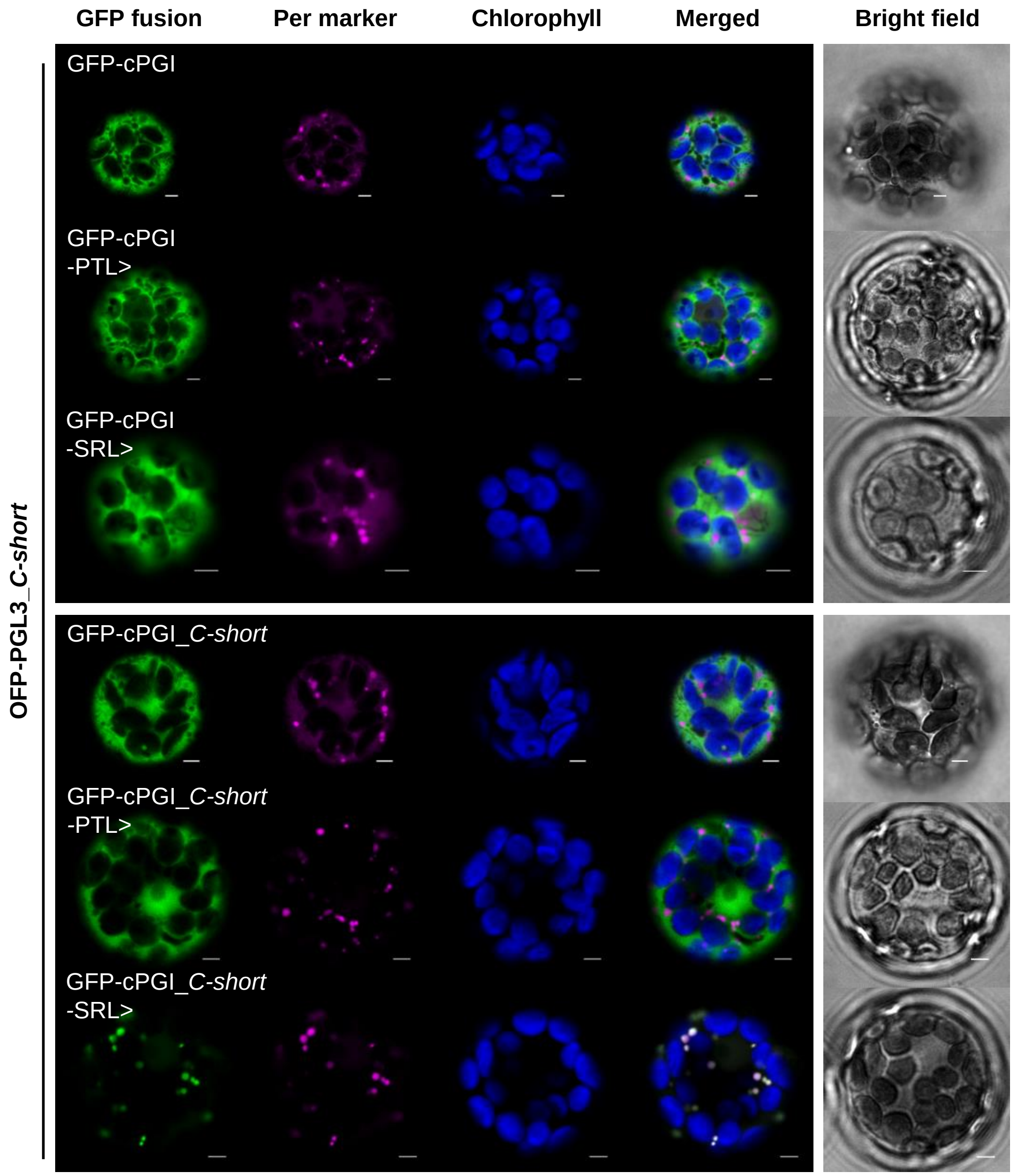

Supplementary Figure S8 (continued). Single channel images of Figure 5B.

Co-expression of peroxisome marker OFP-PGL3_*C-short* (ending -SKL>) with cytosolic glucose-6-phosphate isomerase (cPGI) fused to GFP, as *full-length* version (top panels) or only the last 54 amino acids (*C-short*, bottom panels), ending either wild-typically (-PQM>), with engineered PTS1 motif -PTL> (like G6PD5/6) or -SRL>. The images show single optical sections of the three fluorescent channels. Merged, overlay of all channels; Bright field, cellular reference. GFP in green, OFP in magenta, chlorophyll autofluorescence in blue. White signals in Merged indicate co-localisation or very close signals (<200 nm) of GFP and OFP. Scale bars, 3 µm.

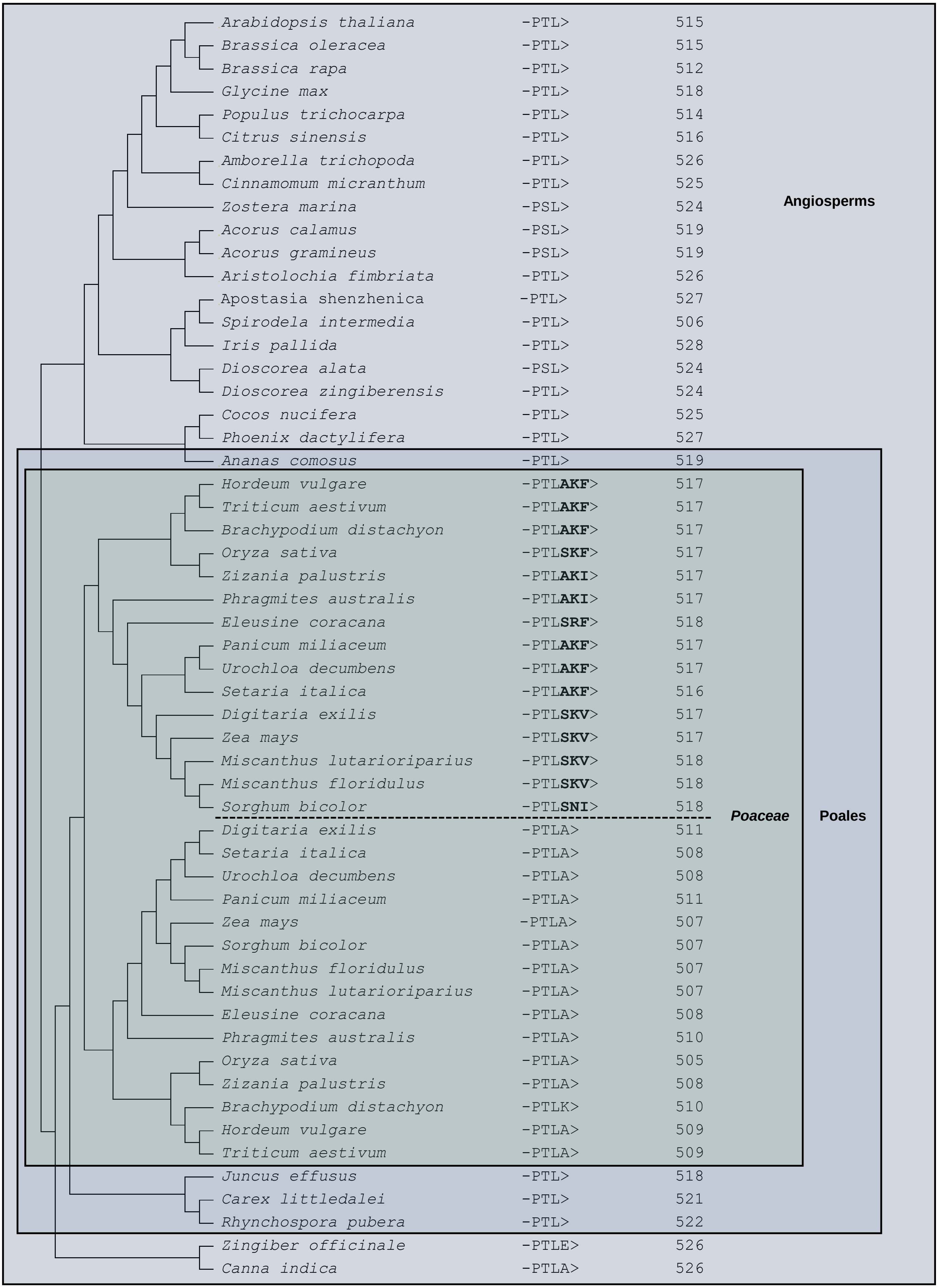

Supplementary Figure S9. More extensive phylogenetic tree with special focus on the family *Poaceae*.
The maximum likelihood tree of AtG6PD6 homologs selected from different species was created with Mega. Subtrees were flipped to present *A. thaliana* on top. The C-terminal 6–9 amino acids highlight conservation of the PTL motif in the Angiosperm sequences with special attention to the differentiation of grasses (*Poaceae*).

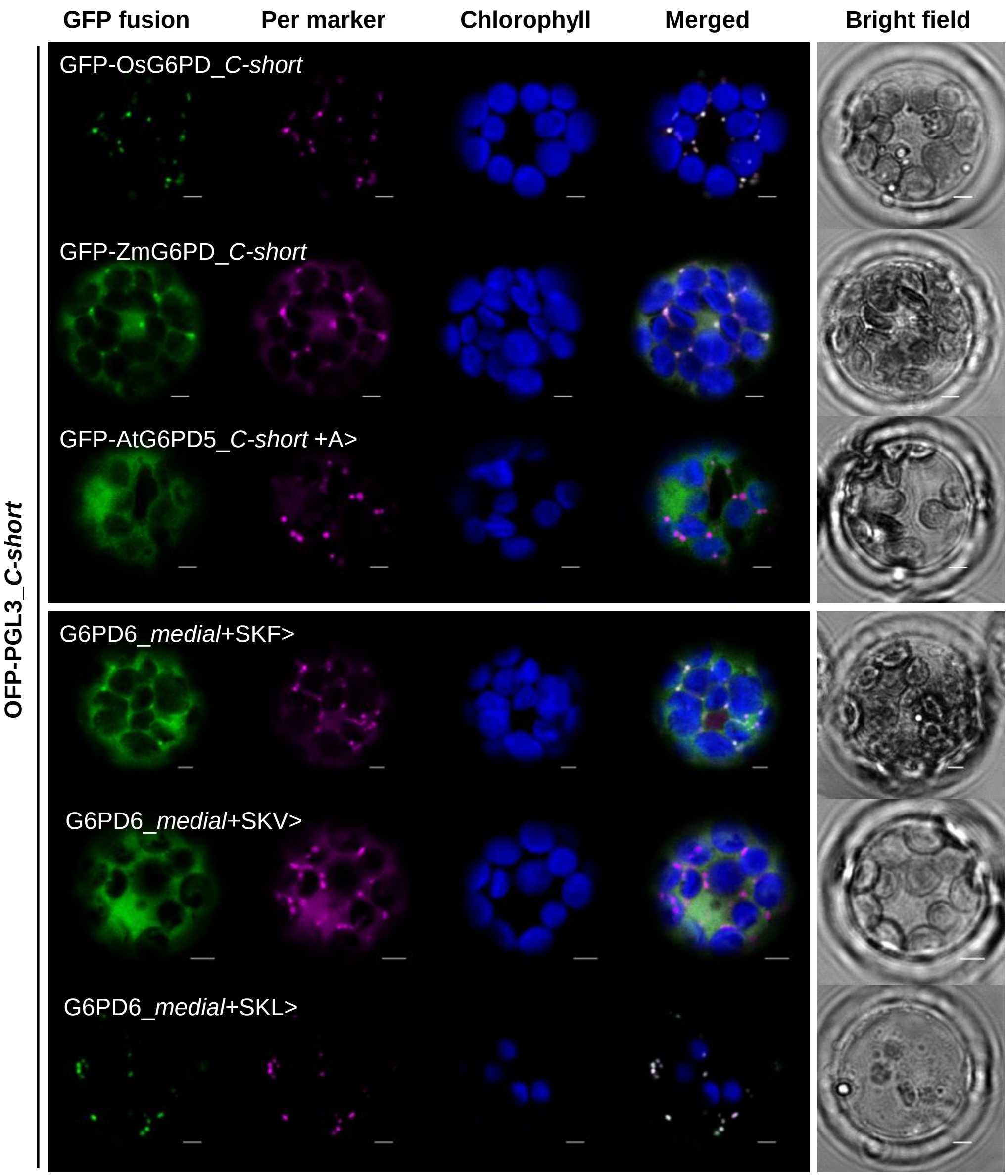

Supplementary Figure S10. Single channel images of Figure 7.

Co-expression of peroxisomal marker OFP-PGL3_*C-short* (ending –SKL>) with *C-short* constructs of G6PD isoforms from rice (Os, *Oryza sativa*), maize (Zm, *Zea mays*), AtG6PD5 with additional alanine (+A), and G6PD6_*medial* ending either like rice (+SKF>), maize (+SKV>), or with attached canonical PTS1 motif (+SKL>). The images show single optical sections of the three fluorescent channels. Merged, overlay of all channels; Bright field, cellular reference. GFP in green, OFP in magenta, chlorophyll autofluorescence in blue. White signals in Merged indicate co-localisation or very close signals (<200 nm) of GFP and OFP. Scale bars, 3 µm.

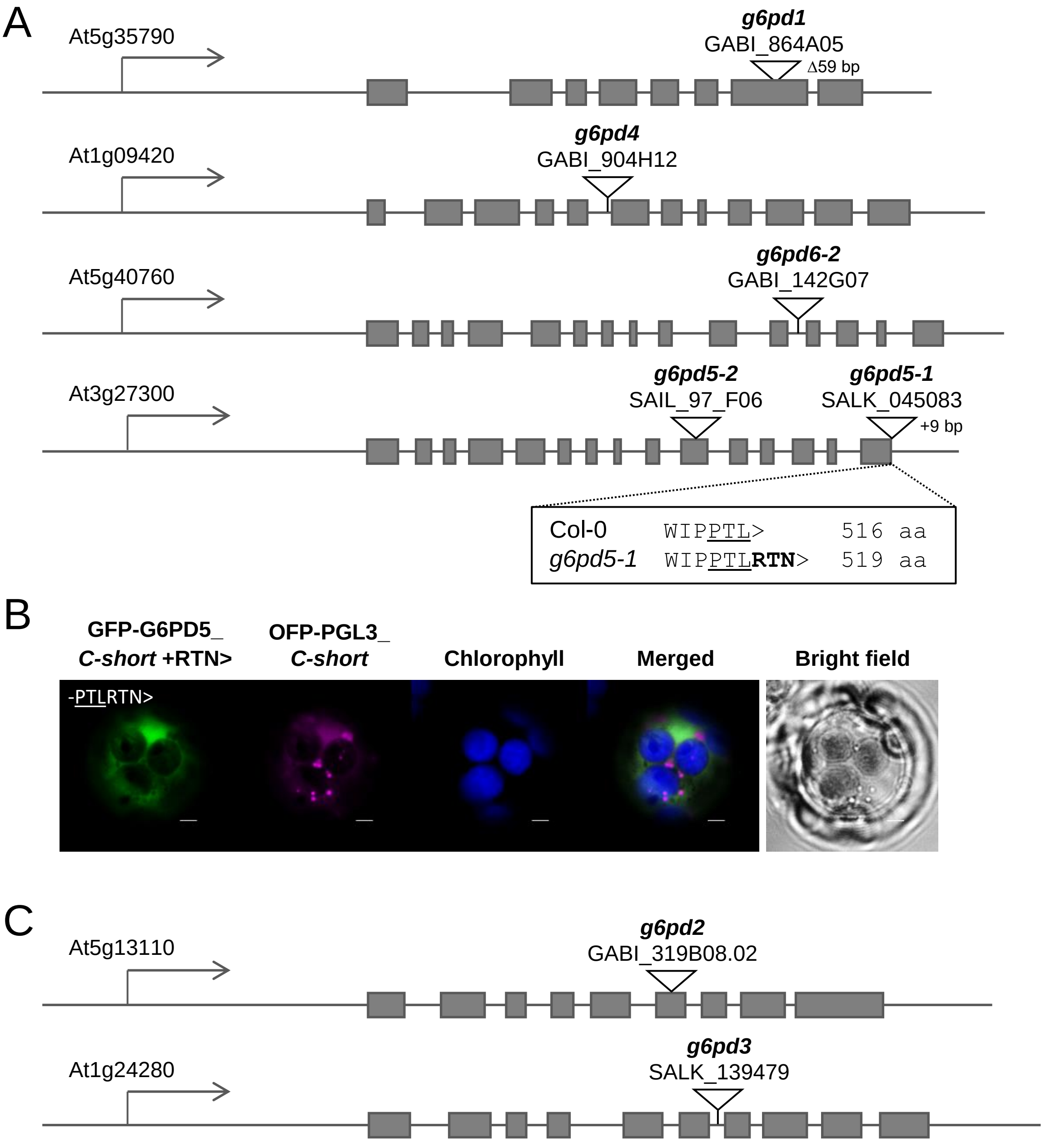

Supplementary Figure S11. Analysis of Arabidopsis T-DNA insertion lines.

A) Intron–exon structure of *G6PD1* (At5g35790), *G6PD4* (At1g09420), *G6PD6* (At5g40760), and *G6PD5* (At3g27300) with positions of relevant T-DNA insertion sites (triangles). Labelling of the *g6pd5-2* mutant allele according to previous reports (Ruan et al. 2022). The C-terminal amino acid sequence resulting from T-DNA insertion in *g6pd5-1* is given below, compared to the wildtype (Col-0). B) Co-expression of peroxisomal marker OFP-PGL3_*C-short* (ending –SKL>) with the GFP *C-short* construct of G6PD5 ending –PTLRTN>, like in the T-DNA mutant allele *g6pd5-1* (panel A). The images show single optical sections of the three fluorescent channels. Merged, overlay of all channels; GFP in green, OFP in magenta, and chlorophyll autofluorescence in blue. Bright field, cellular reference. Scale bars, 3 µm. C) Intron–exon structure of *G6PD2* (At5g13110) and *G6PD3* (At1g24280) with positions of T-DNA insertion sites (triangles).

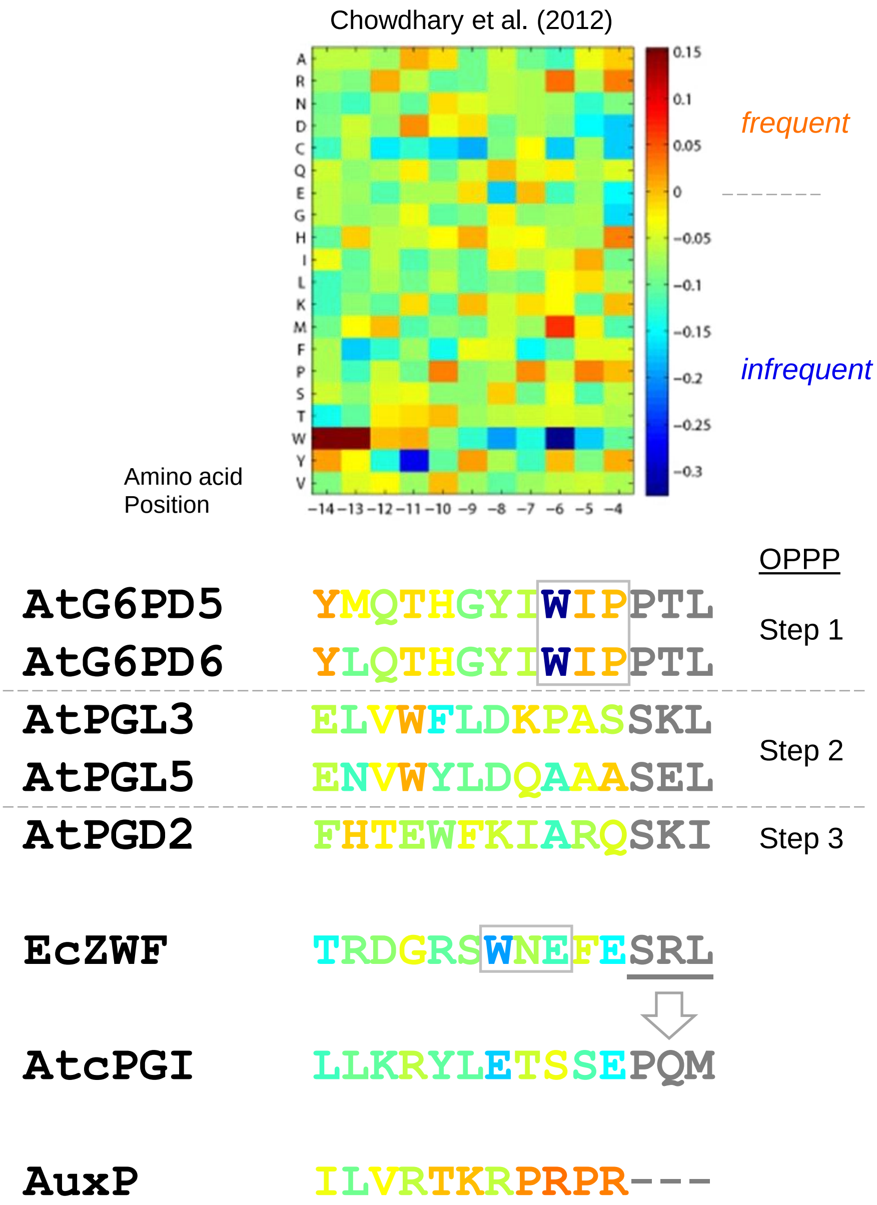

Supplementary Figure S12. Compilation of the PTS1 upstream sequences.

Top, Scheme of Chowdhary et al. (2012) indicating contribution of C-terminal amino acid positions (-4 to -14) upstream of the PTS1 to peroxisomal localization by a colour code: red-orange, good; yellowish, indifferent, green-blue, bad. Centre, upstream sequences of Arabidopsis isoforms contributing to OPPP activity in peroxisomes (G6PD5/6, PGL3/5, and PGD2); PTS1 motifs in grey. The conserved tryptophane (W) at position -6 in G6PD5/6 (also present in *E. coli* ZWF (frames) is suboptimal (dark blue). Note that cytosolic enzymes (EcZWF, AtcPGI) score lowest (mostly amino acids with blue-green colouring). Similar to EcZWF with attached SRL (underlined), engineering cPGI's PQM to SRL (grey arrow) resulted in peroxisome import, yet only of the *C-short* versions (Figures 5, S8). AuxP, auxiliary peptide (position -4 to -6: basic, non-polar, basic amino acid) devised by Deng et al. (2022; 2024) for validating PTS1 motifs, here indicated by grey dashes.
